# Comparative effectiveness of GLP-1 receptor agonists and dual agonists in the treatment of patients with metabolic dysfunction associated steatohepatitis: a systematic review and meta-analysis

**DOI:** 10.3389/fendo.2025.1681965

**Published:** 2025-10-08

**Authors:** Meng Li, Jianli Hu, Yip Han Chin, Han Shi Jocelyn Chew, Wenru Wang

**Affiliations:** ^1^ Endocrinology and Metabolism Department, The First Affiliated Hospital of Xi’an Jiaotong University, Xi’an, Shaanxi, China; ^2^ Alice Lee Centre for Nursing Studies, Yong Loo Lin School of Medicine, National University of Singapore, Singapore, Singapore; ^3^ School of Nursing, Henan University of Science and Technology, Luoyang, Henan, China; ^4^ Ministry of Health Holdings, Singapore, Singapore

**Keywords:** GLP-1 receptor agonists, dual agonists, metabolic dysfunction associated steatohepatitis, hepatic fibrosis, weight loss, cardiovascular disease

## Abstract

**Background:**

Glucagon-like peptide-1 receptor agonists (GLP-1RAs) and dual agonists have been shown to induce histological improvements in patients with metabolic dysfunction-associated steatohepatitis (MASH). However, current clinical evidence on their effectiveness in improving hepatic fibrosis and cardiovascular outcomes remains limited and inconsistent.

**Methods:**

This study synthesized randomized controlled trials (RCTs) from major databases up to August 30, 2025, focusing on patients with biopsy-confirmed MASH. Pooled mean differences were calculated using either a fixed-effects or random-effects model, depending on the degree of heterogeneity observed among the studies.

**Results:**

Six studies including 1,726 participants were analyzed. Compared with placebo, GLP-1RAs and dual agonists significantly increased the likelihood of histological improvement in MASH without worsening hepatic fibrosis. (OR: 4.51, 95% CI: 3.68 to 5.52). It was associated with a ≥1-stage improvement in hepatic fibrosis without worsening MASH (OR: 1.78; 95% CI: 1.47to2.16). In addition, it contributed to MASH resolution accompanied by a ≥1-stage improvement in hepatic fibrosis (OR: 7.42; 95% CI: 2.98to18.48). In subgroup analyses based on post-treatment weight loss, GLP-1RAs and dual agonists demonstrated significant efficacy in promoting hepatic fibrosis resolution without worsening MASH among patients achieving a ≥10% weight loss (OR: 9.59; 95% CI: 4.01to15.18). However, in patients with <10% weight loss, GLP-1RAs and dual agonists did not demonstrate significant differences (OR: 1.30; 95% CI: 0.92to1.83). Moreover, GLP-1RAs and dual agonists achieved a significant pooled reduction in cardiovascular parameters, including total cholesterol (WMD: −4.15 mmol/L; 95% CI: −13.13 to 4.82) and triglycerides (WMD: −17.70 mmol/L; 95% CI: −21.95 to −13.44). Nevertheless, no significant improvement was observed in Low-Density Lipoprotein Cholesterol (LDL-C) levels (WMD: −0.67 mmol/L; 95% CI: −6.55 to 5.21).

**Conclusion:**

In patients with MASH who achieve a ≥10% weight loss, GLP-1RAs and dual agonists are associated with significant improvements in hepatic fibrosis, whereas their effect is limited in those with <10% weight loss. However, a significant reduction in LDL-C was observed only among patients achieving substantial (≥10%) weight loss. This finding suggests that for patients requiring comprehensive cardiovascular risk management, additional lipid-lowering strategies may be needed to optimize the effectiveness of the intervention.

**Systematic Review Registration:**

https://www.crd.york.ac.uk/prospero/, identifier CRD42025640318.

## Study importance

**What is already known?**

- GLP-1RAs and dual agonists have been approved for the treatment of type 2 diabetes and obesity
- These agents significantly reduce body weight, promote MASH resolution, and improve hepatic fibrosis in patients with MASH.
- These agents are associated with improvements in hepatic-related biomarkers, including Aspartate Aminotransferase (AST) and Alanine Aminotransferase (ALT).

**What does this study add?**

- The degree of weight loss plays a crucial role in the improvement of hepatic fibrosis among MASH patients treated with GLP-1RAs and dual agonists.
- Although GLP-1RAs and dual agonists do not directly lower LDL-C in patients with MASH, a significant reduction was observed in those achieving ≥10% weight loss. Conversely, weight loss of <10% was not associated with a reduction in LDL-C.
- This study emphasizes the critical need for monitoring cardiovascular risk in patients with comorbid dyslipidemia.

**How might these results change the direction of research or the focus of clinical practice?**

- The degree of weight loss may serve as a predictor of hepatic fibrosis improvement in patients with MASH.
- Further research is needed in MASH patients with <10% weight loss to determine whether GLP-1RAs and dual agonists provide meaningful improvements in hepatic fibrosis.
- Monitoring LDL-C levels is essential during treatment with GLP-1RAs and dual agonists, with lipid-lowering interventions implemented as needed.

## 1 Introduction

Metabolic dysfunction-associated steatohepatitis (MASH) is an increasingly prevalent contributor to the global burden of liver disease, affecting approximately 5% of adults worldwide (1). Its prevalence is substantially higher among individuals with type 2 diabetes mellitus (T2DM) and obesity, reaching 66.4% and 34%, respectively (1, 2). Advanced hepatic fibrosis is also common in these populations, with stage F3 and F4 fibrosis affecting 11.66% and 1.71%, respectively (3). These findings highlight the high prevalence of MASH in overweight and obese populations and its close association with clinically significant and progressive hepatic fibrosis. MASH is characterized by excessive hepatic fat accumulation, which triggers lipotoxicity and subsequently promotes hepatocellular inflammation and injury. This multifactorial condition is closely associated with metabolic dysfunction, with hepatic steatosis and insulin resistance playing central roles in its pathogenesis (3, 4).The development of hepatic fibrosis in MASH results from persistent hepatocellular inflammation and recurrent hepatocyte damage throughout disease progression (5).This pathological process disrupts the balance between synthesis and degradation of the extracellular matrix (ECM), leading to excessive ECM deposition and ultimately promoting the development of hepatic fibrosis (6).

In recent years, treatment strategies for MASH have advanced considerably, with both lifestyle interventions and pharmacotherapies showing promising efficacy (7–9). Evidence suggests that a 5–7% reduction in body weight can improve hepatic steatosis, whereas a ≥10% weight loss may help reverse hepatic fibrosis (10–12). Moreover, the introduction of both single and multi-target pharmacological agents has markedly expanded the therapeutic landscape for MASH (13). Treatments using glucagon-like peptide-1 receptor agonists (GLP-1RAs), particularly those involving dual agonists, offer distinct therapeutic advantages. Specifically, GLP-1 activation improves insulin sensitivity and promotes weight loss; modulation of glucose-dependent insulinotropic polypeptide receptors (GIPR) reduces hepatic lipogenesis and steatosis; and activation of glucagon receptors (GCGR) enhances energy expenditure and lipid mobilization (14–16). It is also important to emphasize that the precise receptor selectivity of GLP-1 RAs and GLP-1/GIP dual agonists underlies their efficacy and safety. GLP-1RAs demonstrate high specificity for GLP-1R, showing no detectable activity on GIPR or GCGR even at concentrations up to 1 μM (17–19). GLP-1/GIP dual agonists efficiently activate both GLP-1R and GIPR, with potency surpassing that of native GLP-1 and GIP, while inducing only weak activation of GCGR at supraphysiological concentrations and remaining completely unable to antagonize glucagon function. Clinical Positron Emission Computed Tomography (PET) imaging further confirmed that these dual agonists show very low GCGR occupancy in humans (11.2 ± 14.4%), with no statistical significance (17–19).Therefore, GLP-1RAs selectively activate GLP-1R without affecting GIPR or GCGR, whereas GLP-1/GIP dual agonists primarily synergistically activate both GLP-1R and GIPR, with no significant pharmacological activity on GCGR. In addition to their core advantage of precise receptor selectivity, GLP-1 RAs and GLP-1/GIP dual agonists can also provide additional physiological protective effects through other mechanisms. For example, regarding vascular endothelial protection, these agents can mitigate oxidative stress by inhibiting the activation of the Nicotinamide Adenine Dinucleotide Phosphate Hydrogen (NADPH) oxidase complex, specifically via downregulation of Nicotinamide Adenine Dinucleotide Phosphate Oxidase 4(NOX4) expression and blockade of p47^phox translocation. They can also suppress the expression of adhesion molecules, such as Vascular Cell Adhesion Molecule-1(VCAM-1), thereby reducing inflammatory responses and providing multifaceted protection to the vascular endothelium (20, 21). Such multi-target strategies offer a promising approach to address the complex pathophysiology of MASH.

Despite growing interest in GLP-1RAs and dual agonists, current clinical trial evidence remains inconsistent. In particular, their efficacy and safety in improving hepatic fibrosis are unclear, as is their impact on cardiometabolic markers. While reductions in total cholesterol and triglycerides have been observed, effects on Low-Density Lipoprotein Cholesterol (LDL-C) remain uncertain. Although recent MASH management guidelines recognize (22) the potential of dual agonists in reducing disease activity and mitigating hepatic fibrosis worsening, conclusive histological evidence for hepatic fibrosis reversal is still lacking (22). Given the critical knowledge gaps outlined above, this meta-analysis aims to: (1) synthesize existing evidence to evaluate the histological efficacy of GLP-1RAs and dual agonists in MASH; (2) assess the dose–response relationship on treatment outcomes; and (3) systematically examine modulating cardiometabolic parameters, thereby clarifying their therapeutic potential in mitigating residual cardiovascular risk, as dysregulated cardiometabolic parameters are recognized contributors to atherosclerotic progression in patients with MASH.

## 2 Methods

### 2.1 Study design

The protocol for this meta-analysis was registered with PROSPERO (CRD42025640318). The study was conducted in accordance with the Preferred Reporting Items for Systematic Reviews and Meta-Analyses (PRISMA) guidelines. The PRISMA checklist was adhered to throughout the review to ensure methodological transparency and consistency (23).

This study employs a systematic review approach to synthesize findings from multiple RCTs, aiming to provide a more accurate estimate of the overall treatment effect of GLP-1RAs and dual agonists on hepatic histology and cardiovascular parameters in patients with MASH, and to explore the influence of weight change on their efficacy.

### 2.2 Literature search strategy

Electronic databases, including the Cochrane Library, Embase, MEDLINE (via PubMed), CENTRAL, and Web of Science, were systematically searched. The initial search was conducted on July 21, 2024, and subsequently updated on August 30, 2025, to identify RCTs evaluating the effects of GLP-1 RAs and dual agonists in the treatment of MASH. Key search terms such as “Non-alcoholic Fatty Hepatic Disease,” “Nonalcoholic Steatohepatitis,” “MASH,” “GLP-1 receptor agonist,” “semaglutide,” “dulaglutide,” “dual agonists,” “Gastric Inhibitory Polypeptide,” and “Glucose Dependent Insulinotropic Peptide” were used in the search strategy. A detailed description of the search strategy, including the specific search strings, is provided in **Supplementary Materials 2**. Reference lists from relevant systematic reviews and included studies were also screened for studies to be included. To ensure that all relevant articles were included, searches were conducted in the European Association for the Study of Diabetes (EASD), American Diabetes Association (ADA), International Diabetes Federation (IDF), American Association for the Study of Liver Diseases (AASLD), and ClinicalTrials.gov for other potential studies to be included.

### 2.3 Inclusion and exclusion criteria

The inclusion criteria for the studies were as follows: (1) MASH diagnosis confirmed by hepatic biopsy at enrollment; (2) randomized controlled trial (RCT) design; (3) comparison of the efficacy of GLP-1RAs and dual agonists versus placebo in patients with MASH; (4) treatment duration of at least 24 weeks; and (5) primary efficacy endpoints assessed by repeat hepatic biopsy. The exclusion criteria were as follows: (1) repeated publications; (2) *post hoc* analysis; (3) articles that did not report main outcome measures defined in this article; and (4) articles that did not provide needed data; (5) all observational studies, reviews, meta-analyses, conference proceedings, editorials, commentaries, and unpublished articles; (6) For studies with overlapping participant pools or data sources, only the most comprehensive papers were selected. Hepatic biopsy has been regarded as the gold standard for diagnosing and staging steatohepatitis and hepatic fibrosis, particularly in patients with unclear clinical symptoms. Therefore, only studies with hepatic biopsy-proven MASH were included (22). MASH is defined as hepatic steatosis (HS) involving ≥5% of hepatic tissue, accompanied by hepatocellular inflammation and injury (e.g., hepatocyte ballooning), with or without hepatic fibrosis. Diagnosis requires hepatic histopathological examination and the exclusion of similar pathological changes attributable to other established etiologies (e.g., viral hepatitis, drug-induced hepatocellular injury) (24, 25).

### 2.4 Data extraction

**Figure 1:**
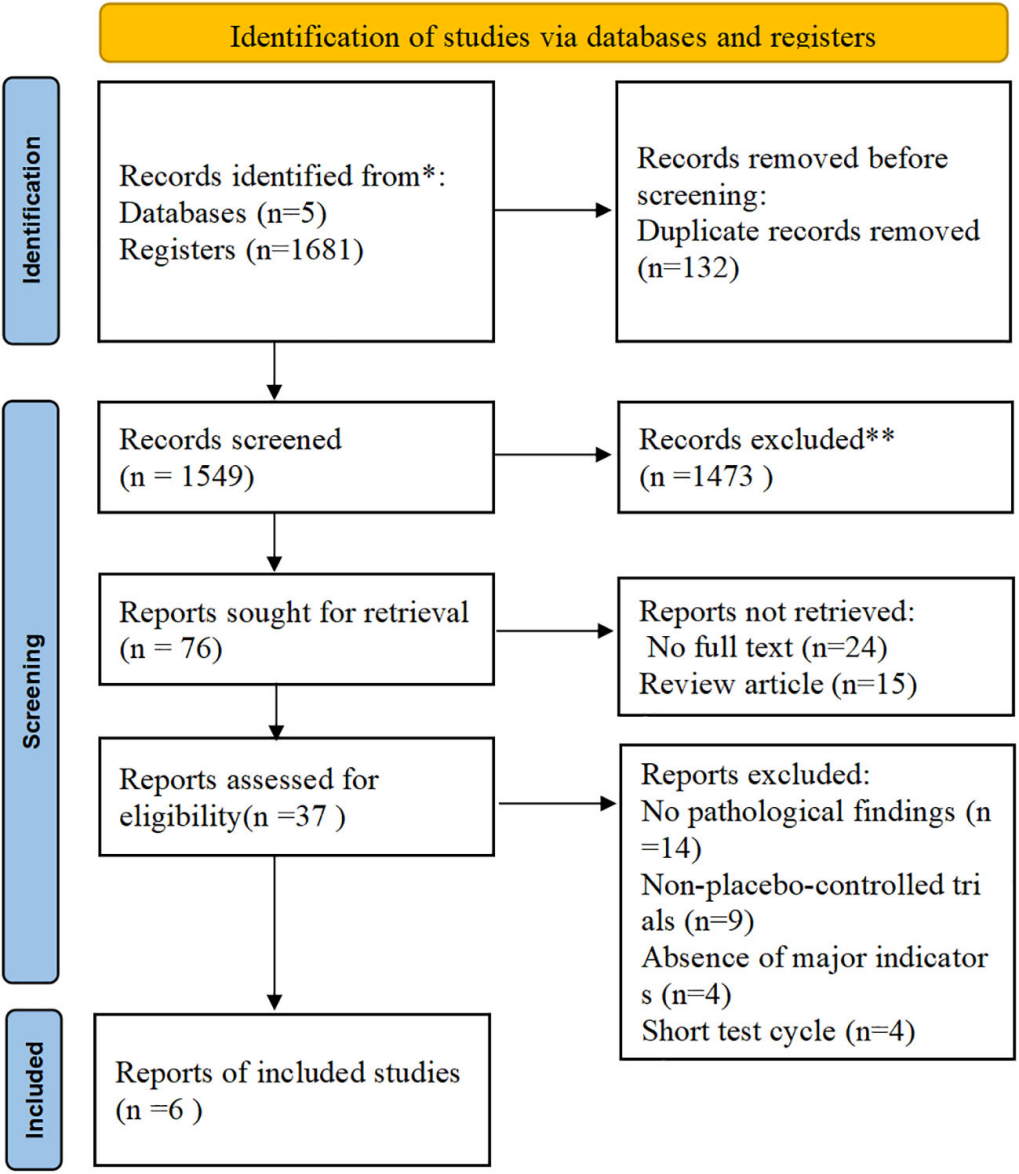
Preferred reporting items for systematic reviews and meta-analyses (PRISMA) flow diagram. (Source: Page MJ, et al. BMJ 2021;372: n71. doi: 10.1136/bmj.n71. This work is licensed under CC BY 4.0. To view a copy of this license, visit https://creativecommons.org/licenses/by/4.0/).

All identified records were imported into EndNote (version 21.5) for deduplication. Two independent reviewers performed the study selection and data extraction. Any conflicts in screening or extraction were resolved through discussion or, when necessary, by referral to a third reviewer. A PRISMA flowchart visually depicts the study selection process (**Figure 1**). Data were extracted into a pre-determined data sheet, which focused on participant demographics (age, gender, body mass index [BMI], body weight, and comorbidity with type 2 diabetes), study characteristics (author, country, type of study, type of funding, and duration of follow-up), and intervention details (including drug names, dosages, and treatment duration). Relevant primary and secondary outcomes were also recorded. The primary outcomes extracted from the included studies were as follows: resolution of MASH without worsening of hepatic fibrosis, an improvement with ≥1-stage hepatic fibrosis without worsening of MASH, and concurrent resolution of MASH with ≥1-stage improvement in hepatic fibrosis. The secondary outcomes extracted from the included studies were as follows: changes in lipid profiles (e.g., LDL-C, Total Cholesterol [TC], and Triglycerides [TG]), hepatic enzyme levels (e.g., Alanine Aminotransferase [ALT] and Aspartate Aminotransferase [AST]), and body weight. Adverse event outcomes included Gastrointestinal (GI) events such as nausea, vomiting, and diarrhea. In the context of existing reviews, the resolution of MASH is defined as the complete absence of hepatic steatosis and ballooning, mild or no inflammatory changes, and no progression in the NAFLD Activity Score (NAS). Consequently, “an improvement of ≥1-stage hepatic fibrosis without worsening of MASH” is defined by three criteria: (1) a ≥1-stage reduction in hepatic fibrosis; (2) no worsening of steatosis, ballooning, or lobular inflammation; and (3) no progression in the NAS (26, 27, 69).

### 2.5 Risk of bias assessment

**Figure 2:**
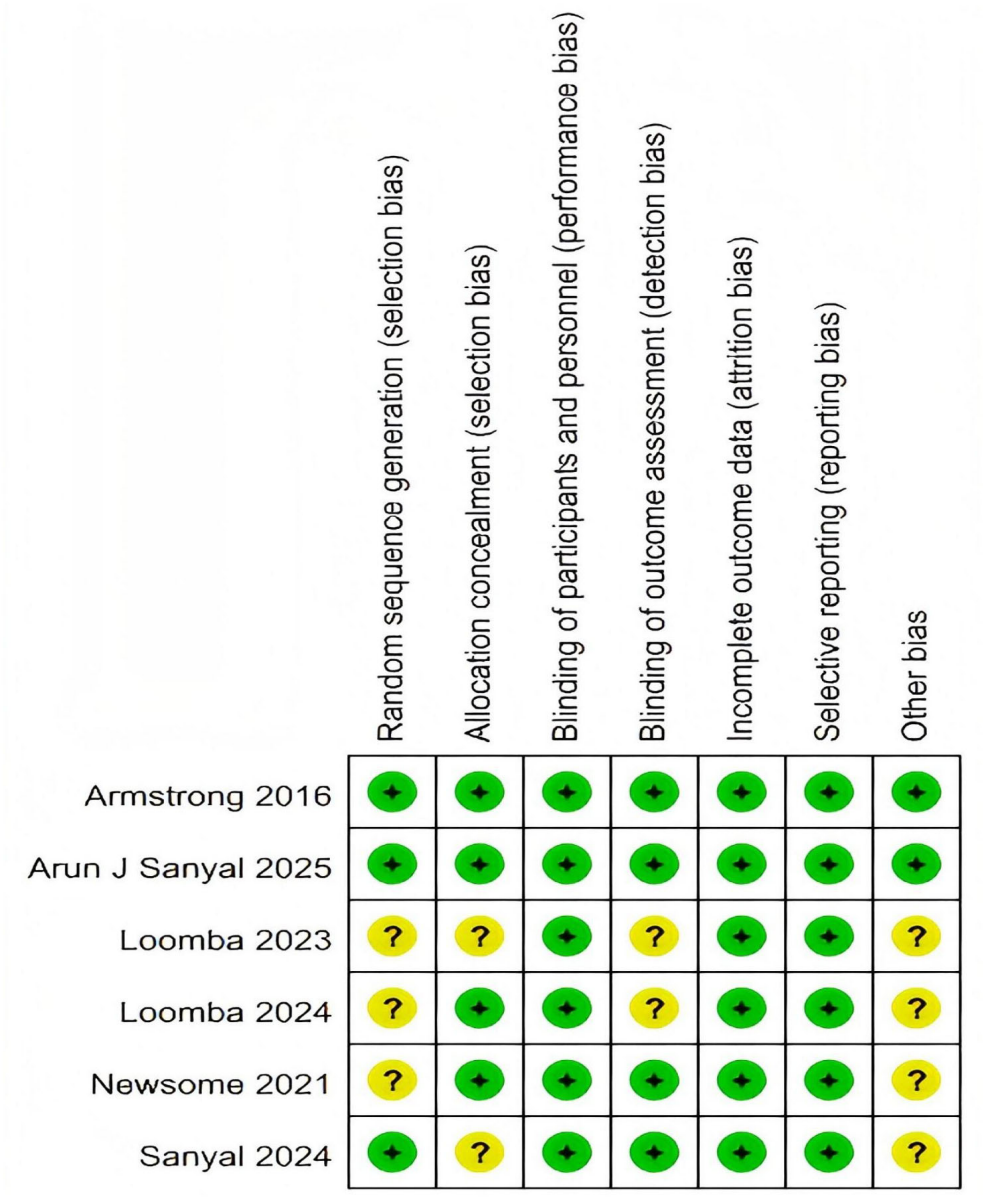
Risk of bias summary of studies included in the meta-analysis (Cochrane RoB 2.0 tool).

The risk of bias for each included study was independently assessed by two researchers, who were not involved in the studies, using the Cochrane Risk of Bias Tool 2.0 (RoB-2) (28). This tool evaluates bias across six domains: selection bias, performance bias, detection bias, attrition bias, reporting bias, and other potential sources of bias. Judgments were categorized as low risk, some concerns, or high risk of bias. All included studies were rated as high quality, with a low risk of bias (**Figure 2**, **Supplementary Material 3**).

### 2.6 Statistical analysis

Statistical analyses were conducted using Stata version 18 and Review Manager (RevMan) version 5.4.1 (29, 30). A meta-analysis was conducted by pooling data from the included RCTs to compare the effectiveness of GLP-1RAs and dual agonists in resolving MASH and improving hepatic fibrosis. Pooled Odds Ratios (OR) were computed across the studies. If the I² value was no more than 50% and P > 0.05, indicating that heterogeneity was negligible, the fixed-effect model was used; otherwise, a random-effects model was used to pool the data (31). Standard Deviation (SD) is required for performing the meta-analysis. However, most of the studies included in this meta-analysis did not provide SDs but only a Standard Error (SE) or a 95% Confidence Intervals (CIs). Therefore, we employed a formula outlined in the Cochrane Handbook for Systematic Reviews of Interventions to calculate SDs (32).

Effect sizes and their corresponding 95% CIs were extracted from the primary trial reports and any additional data sources, including **Supplementary Appendices**. For dichotomous outcomes, ORs were computed (e.g., for the primary outcome and GI adverse events), while Weighted Mean Differences (WMDs) were used for continuous outcomes (e.g., body weight), with both accompanied by 95% CIs. Heterogeneity between included studies was evaluated using the I Squared(I ^2^) statistic and visual inspection of forest plots (33, 34).

## 3 Results

### 3.1 Characteristics of included studies

A total of 1,681 publications were identified through the initial search. After title and abstract screening, 76 articles were deemed eligible for full-text review. Of these, 37 underwent full-text assessment, and ultimately, six RCTs involving 1,726 participants were included in the analysis (35–40). Of the included studies, participants received once-daily subcutaneous injections of liraglutide (GLP-1RAs) at a dose of 1.8 mg, with an intervention period of 48 weeks (35). In other studies, participants were administered once-daily subcutaneous semaglutide (GLP-1RAs) at doses of 0.1 mg, 0.2 mg, or 0.4 mg, with an intervention period of 72 weeks (36). Furthermore, both studies employed once-weekly subcutaneous semaglutide at a dose of 2.4 mg, with intervention periods of 48 weeks (37) and 72 weeks (40). In addition, participants were administered once weekly subcutaneous tirzepatide (GLP-1/GCG dual agonist) at doses of 5 mg, 10 mg, or 15 mg, with an intervention period of 52 weeks (38), or once weekly subcutaneous survodutide (GLP-1/GIP dual agonist) at doses of 2.4 mg, 4.8 mg, or 6.0 mg, with an intervention period of 48 weeks (39). All studies were phase II trials, except for the study by Arun J. Sanyal (40), which was a phase III trial of semaglutide (35, 39). Specifically, there were six multicenter studies, with North America, Europe, and the Western Pacific being the top regions where the RCTs were conducted. The mean age of participants was 54.39 ± 11.03 years, and the mean BMI was 35.55 ± 6.25 kg/m². The study population comprised 43% males and 57% females. Among MASH patients, 35.63% were classified as F2 and 55.85% as F3 for hepatic fibrosis stage. In addition, 50% of participants had comorbid T2DM, and the average intervention duration was approximately 57 weeks (**Table 1**).

**Table 1:** Baseline characteristics of the included studies.

Study	Patient characteristics	Dose and duration	Primary outcome	Adverse events
1. Armstrong (35) et al. (2016) UK	average age of patients with hepatic biopsy confirmed MASH and hepatic fibrosis: 51 years; 60% male; BMI: 35.9 ± 5.4; percentage of comorbid diabetes mellitus: 32%; ALT: 71 IU/L; AST: 51 IU/L; F0 -F2 (histologically) 48%; hepatic fibrosis F3 -F4 (histologically) 52%; TC: 173mg/dL; TG: 168 mg/dL; LDL-C: 100 mg/dL	A. Liraglutide 1.8 mg/day (n = 26) B. Placebo (n = 26) Duration of intervention: 48 weeks	MASH histologic resolution: 39% vs 9% p=0.019; improvement in hepatic tissue hepatic fibrosis by 1 point or more with no worsening of MASH: 26% vs 14% p=0.46; change from baseline in ALT: -26.6 vs -10.2UI/L, p = 0.16; change in AST: -15.8 vs -8.6IU/L; p = 0.29; change in weight: -5.3 vs -0.6kg, p = 0.003	The incidence of adverse events in the liraglutide treated and placebo groups, was nausea (46% vs. 38%), diarrhea (38% vs. 19%), and vomiting (19% vs. 12%).
2. Newsome (36) et al. (2020) International cohort of individuals from 16 countries	average age of patients with hepatic biopsy confirmed MASH and hepatic fibrosis: 55 years; 339% male; body weight:97.37zf521.07; BMI: 35.7 ± 6.4; percentage of comorbid diabetes mellitus: 37%; ALT:54IU/L; AST: 43IU/L; F2-F3 (histologically) 57%; TC: 186mg/dL; TG: 167mg/dL; LDL-C: 105mg/dL	A. Semaglutide 0.1 mg/day (n = 80) B. Semaglutide 0.2 mg/day (n = 78) C. Semaglutide 0.4 mg/day (n = 82) D. Placebo (n = 80) Duration of intervention: 72 weeks	The proportion of patients with NASH resolution without worsening of hepatic fibrosis was 40% in the 0.1 mg group, 36% in the 0.2 mg group, 59% in the 0.4 mg group, and 17% in the placebo group (*p* < 0.001 for 0.4 mg vs. placebo). The proportion of hepatic tissue hepatic fibrosis improving by 1 point or more with no worsening of MASH was 49% in the 0.1 mg group, and 32% in the 0.2 mg group. 43% in the 0.4 mg group and 33% in the placebo group (0.4 mg vs. placebo *p* = 0.48). Thirty seven percent of patients in the 0.4 mg group and 15% of patients in the placebo group experienced resolution of MASH and improved hepatic fibrosis stage. Treatment with Semaglutide resulted in a dose dependent reduction in serum ALT and AST levels, with a average weight loss of 13% in the 0.4 mg group and 1% in the placebo group (*p* < 0.001).	The incidence of adverse events in the survodutide treated and placebo groups, was nausea (36% vs. 11%), diarrhea (25% vs. 14%), and vomiting (17% vs. 2%).
3. Loomba (37) et al. (2023) International cohort of individuals from 38 countries	The average age of patients with hepatic biopsy confirmed MASH and hepatic fibrosis: 59 years old; 31% male; body weight: 95.2 ± 19.7; BMI: 35 ± 5.9; percentage of comorbid diabetes mellitus: 74%; ALT: 42 IU/L; AST: 43 IU/L; TC: 177 mg/dL; TG: 168 mg/dL; LDL-C: 100 mg/dL	A. Semaglutide 2.4 mg/day (n = 47) B. Placebo (n = 24) Length of intervention: 48 weeks	NASH resolution without worsening of hepatic fibrosis:34% VS 21%; p=0.29; improvement of hepatic tissue hepatic fibrosis by 1 point or more with no worsening of MASH: 11% VS 29% p=0.087; ALT: -20.2 VS 1.9 IU/L, p=0.009; AST: -16.0 VS 1.5 IU/L; change in body weight: -8.83 VS - 0.09kg/m2; p<0.0001;	The incidence of adverse events in the liraglutide treated and placebo groups, was nausea (45% vs. 17%), diarrhea (19% vs. 8%), and vomiting (17% vs. 0%).
4. Loomba (38) et al. (2024) International cohort of individuals from 10 countries	The average age of patients with hepatic biopsy confirmed MASH and hepatic fibrosis was 54 years; 43% male; body weight:99.8 ± 21.5; BMI: 36.1 ± 6.1; the proportion of comorbid diabetes mellitus: 42%; ALT:61IU/L; AST: 50IU/L; TG: 171mg/dL; LDL-C: 106mg/dL	A. Tirzepatide 5mg/day (n=47) B. Tirzepatide 10mg/day (n=47) C. Tirzepatide 15mg/day (n=48) D. Placebo (n=48) Length of intervention: 52weeks	Proportion of patients with MASH resolution without worsening of hepatic fibrosis 44% in the 5mg group; 56% in the 10mg group; 62% in the 15mg group; and 10% in the placebo group; (*p* < 0.001 for 15mg vs. placebo); Proportion of patients with improvement in hepatic tissue hepatic fibrosis of 1 point or more with no worsening of MASH 55% in the 5mg group; 51% in the 10mg group; 51% in the 15mg group ( 15mg vs. placebo *p* < 0.001); 30% in placebo group; Proportion of patients with improvement in MASH with concomitant improvement in hepatic tissue hepatic fibrosis of 1 point or more 55% in the 5mg group; 51% in the 10mg group; 51% in the 15mg group; and 30% in the placebo group (*p* < 0.001 for 15mg vs. placebo); ALT change:-51.6 in the 5mg group vs. 5.6UI/L in the 5mg group, -56.0 vs -5.6UI/L in the 10mg group; -56.7 vs -5.6UI/L in the 15mg group, all *p* < 0.01; AST change: -42.1 vs -3.8UI/L in the 5mg group, -47.7 vs -3.8UI/L in the 10mg group; -47.1 vs -3.8UI/L in the 15mg group, all *p* < 0.01; ALT change:-42.1 vs -3.8UI/L in the 5mg group, 47.7 vs -3.8UI/L in the 10mg group; -47.1 vs -3.8UI/L in the 15mg group, all *p* < 0.01; body weight change: 5mg group -10.7 VS -0.8kg, 10mg group -13.3 VS --0.8kg; 15mg group -15.6 VS -0.8kg, *p* < 0.01	The incidence of adverse events in the liraglutide treated and placebo groups, was nausea (38% vs. 12%), diarrhea (32% vs. 23%), and vomiting (9% vs. 2%).
5. Sanyal (39) et al. (2024) International cohort of individuals from 25 countries	The average age of patients with hepatic biopsy confirmed MASH and hepatic fibrosiswas 50 years; 47% male; bodyweight:100.84 ± 22.37; BMI: 35.8 ± 6.4; the proportion of combined diabetes mellitus: 61%; ALT:57UI/L; AST:47UI/L	A. survodutide 2.4mg/day (n=73) B. survodutide 4.8mg/day (n=72) C. survodutide 6.0mg/day (n=74) D. Placebo (n=74) Duration of intervention: 48weeks	In the 2.4 mg, 4.8 mg and 6.0 mg, and placebo treatment groups, the percentages of participants with improved MASH and no worsening of hepatic fibrosis were 47%, 62%, 43%, and 14%, respectively; the proportions of patients with an improvement of 1 point or more in hepatic tissue hepatic fibrosisand no worsening of MASH were 34% in the 2.4 mg group, 36% in the 4.8 mg group, 34% in the 6.0 mg group, and 39.0% in the placebo group 22%; the proportion of patients with improvement in MASH and concomitant improvement in hepatic tissue hepatic fibrosis of 1 point or more was 42.5% in the 2.4 mg group; 47.2% in the 4.8 mg group; 39.2% in the 6.0 mg group; and 5.4% in the placebo group; and the change in ALT was:-35.4 vs -5.7 UI/L in the 2.4 mg group, -38.5 vs -5.7 UI/L in the 4.8 mg group; -38.5 vs -5.7 UI/L in the 6.0 mg group -38.5 VS -5.7UI/L, all *p* < 0.01; AST change: 2.4mg group -27.5 VS --2.4UI/L, 4.8mg group -28.3 VS --2.4UI/L; 6.0mg group -32.2 VS -2.4UI/L, *p* < 0.01; weight change: 2.4mg group -10.03 VS -- 0.89kg, 4.8mg group -12.96 VS --0.89kg; 6.0mg group -13.82 VS -0.89kg, *p* < 0.01	The incidence of adverse events in the survodutide treated and placebo groups, was nausea (66% vs. 23%), diarrhea (49% vs. 23%), and vomiting (41% vs. 4%).
6. Arun J. Sanyal, (40) et al. (2025) International cohort of individuals from 37 countries	The average age of patients with hepatic biopsy confirmed MASH and hepatic fibrosis was 55years; 43% male; bodyweight:96.5 ± 24.55; BMI: 34.65 ± 7.15; the proportion of combined diabetes mellitus: 56%; ALT:67UI/L; AST:53UI/L	A. Semaglutide 2.4 mg/day (n = 534) B. Placebo (n = 266) Length of intervention: 72 weeks	In the 2.4 mg and placebo treatment groups, resolution of steatohepatitis without worsening of fibrosis occurred in 62.9% of the 534 patients in the semaglutide group and in 34.3% of the 266 patients in the placebo group (estimated difference, 28.7 percentage points; 95% CI, 21.1 to 36.2; P < 0.001). A reduction in liver fibrosis without worsening of steatohepatitis was observed in 36.8% of patients in the semaglutide group and in 22.4% of those in the placebo group (estimated difference, 14.4 percentage points; 95% CI, 7.5 to 21.3; P < 0.001). Additionally, the change in ALT was -52.1 U/L vs. -22.2 U/L in the 2.4 mg group (P < 0.01), the change in AST was -44.9 U/L vs. -17.1 U/L (P < 0.01), and the weight change was -10.05 kg vs. -2.0 kg (P < 0.01).	The incidence of adverse events in the survodutide treated and placebo groups, was nausea (36% vs. 13%), diarrhea (27% vs. 12%), and vomiting (19% vs. 6%).

### 3.2 Effect of GLP-1RAs and dual agonists on primary outcome

**Figure 3:**
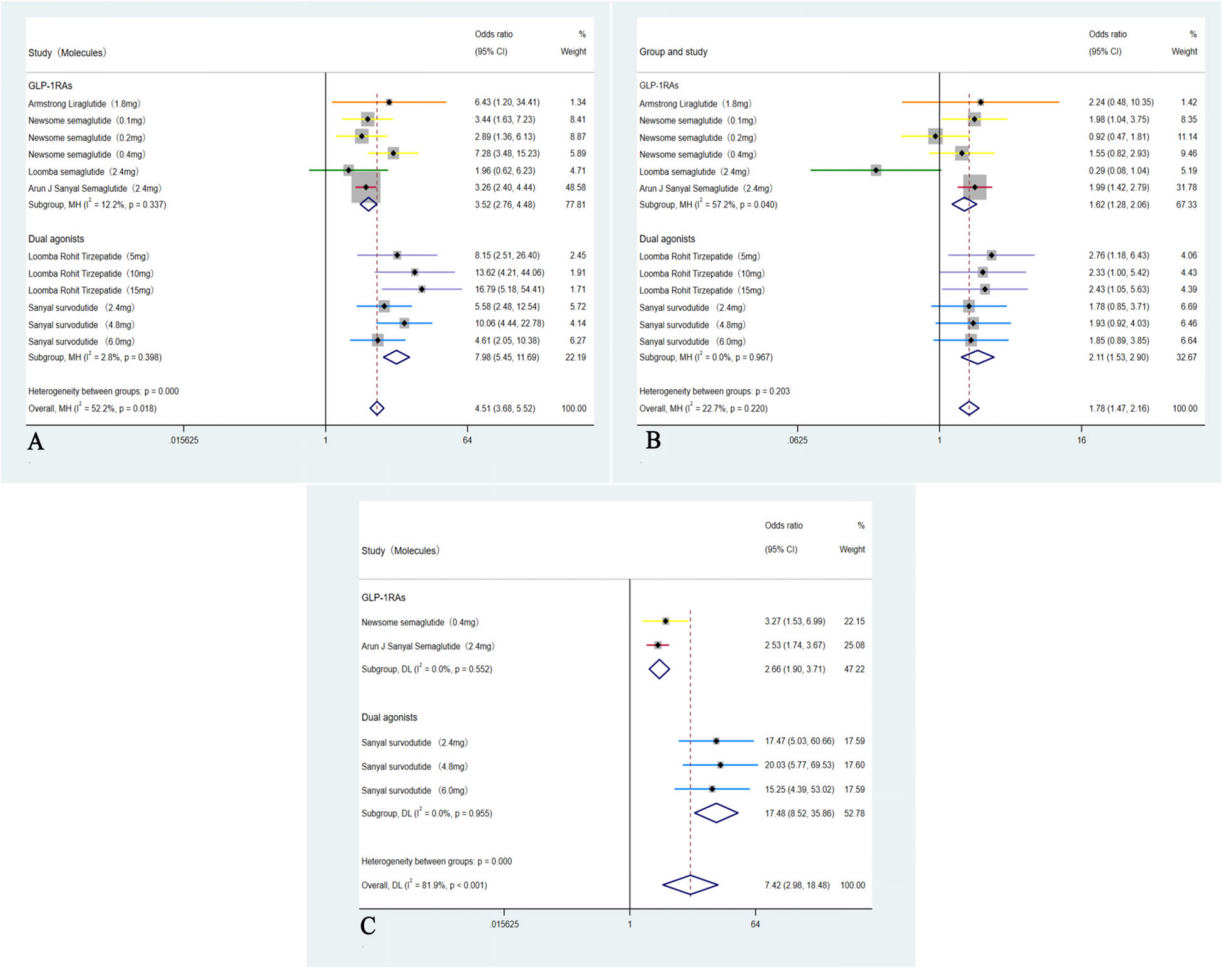
Analysis of primary outcomes: **(A)** Forest plot of ORs for resolution of MASH without worsening of hepatic fibrosis; **(B)** Forest plot of ORs for improvement in hepatic fibrosis by ≥1-stage without worsening of MASH; **(C)** Forest plot of ORs for concurrent resolution of MASH and ≥1-stage improvement in hepatic fibrosis.

A total of six RCTs including 1,726 participants were incorporated into the analysis (35–40). Compared with placebo, GLP-1RAs and dual agonists were associated with an OR of 4.51 (95% CI: 3.68 to 5.52; I² = 52.2%; *p* < 0.01) for achieving MASH resolution without worsening of hepatic fibrosis. The ORs for improvement in hepatic fibrosis by ≥ 1-stage without worsening of MASH was 1.78 (95% CI: 1.47 to 2.16; I² = 22.7%; *p* < 0.01). For achieving both MASH resolution and a ≥ 1-stage improvement in hepatic fibrosis, the OR was 7.42 (95% CI: 2.98 to 18.48; I² = 81.9%; *p* < 0.01) (**Figure 3**).

#### 3.2.1 Subgroup analyses

**Figure 4:**
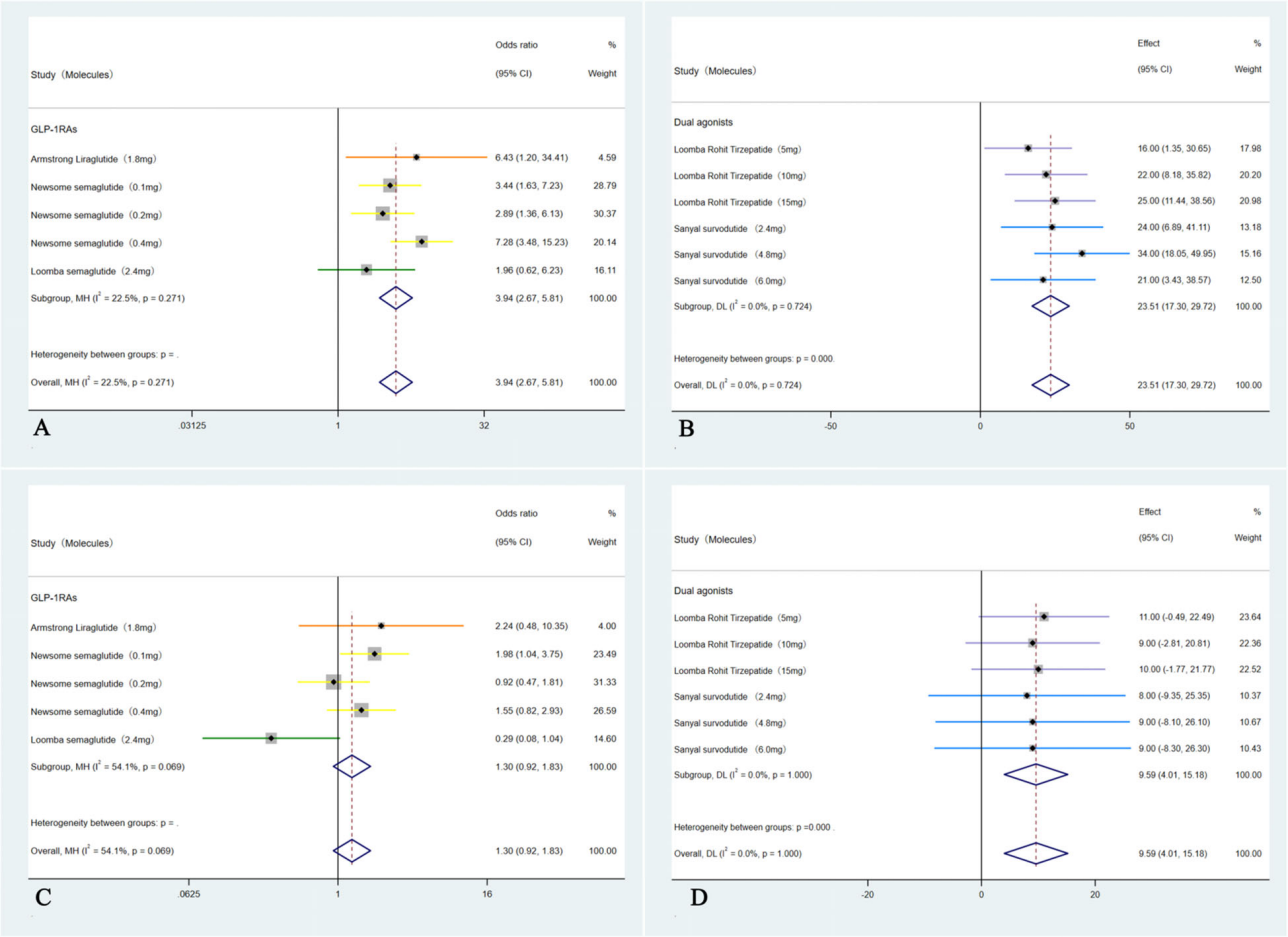
Subgroup analyses of primary outcomes were conducted by categorizing patients according to weight loss: <10% weight loss (GLP-1RAs) and ≥10% weight loss (Dual agonists). The analyses included: **(A)** Forest plot of ORs for MASH improvement without worsening of hepatic fibrosis in the <10% weight loss (GLP-1RAs) group; **(B)** Forest plot of ORs for MASH improvement without worsening of hepatic fibrosis in the ≥10% weight loss (Dual agonists) group; **(C)** Forest plot of ORs for hepatic fibrosis improvement by ≥1 stage without worsening of MASH in the <10% weight loss (GLP-1RAs) group; and **(D)** Forest plot of ORs for hepatic fibrosis improvement by ≥1 stage without worsening of MASH in the ≥10% weight loss (Dual agonists) group.

These five studies (35–39) were analyzed to further examine the impact of weight loss on the primary outcomes; subgroup analyses were conducted based on weight loss categories: <10% (GLP-1RAs) and ≥10% (Dual agonists). For the outcome of MASH resolution without worsening of hepatic fibrosis, the <10% weight loss group (GLP-1RAs) showed an OR of 3.94 (95% CI: 2.67 to 5.81; I² = 22.5%; *p* < 0.01), whereas the ≥10% weight loss group (Dual agonists) demonstrated a higher OR of 23.51 (95% CI: 17.30 to 29.72; I² =0.0%; *p* < 0.01). Regarding the outcome of ≥1-stage improvement in hepatic fibrosis without worsening of MASH, the <10% weight loss group (GLP-1RAs) showed an OR of 1.30 (95% CI: 0.92 to 1.82; I² = 54.1%; *p* = 0.14), indicating no statistically significant difference. In contrast, the ≥10% weight loss group (Dual agonists) demonstrated a significant improvement, with an OR of 9.59 (95% CI: 4.01to 15.18; I² = 0.0%; *p* < 0.01).Except for the non-significant result observed in the <10% weight loss group (GLP-1RAs) for ≥1-stage improvement in hepatic fibrosis without worsening of MASH, all other endpoints across the respective groups showed statistically significant differences (*p* < 0.01). Subgroup analyses for the outcome of achieving both MASH resolution and a ≥1-stage improvement in hepatic fibrosis could not be performed due to insufficient data. Furthermore, although the study by Arun J. Sanyal et al. (2025) (40) used semaglutide 2.4 mg (a GLP-1RA) and reported weight loss of ≥10%, the relevant outcome data could not be analyzed separately. Consequently, this study was included only in the overall pooled analysis and not in any subgroup analyses (**Figure 4**).

### 3.3 Effect of GLP-1RAs and dual agonists on secondary outcomes

**Figure 5:**
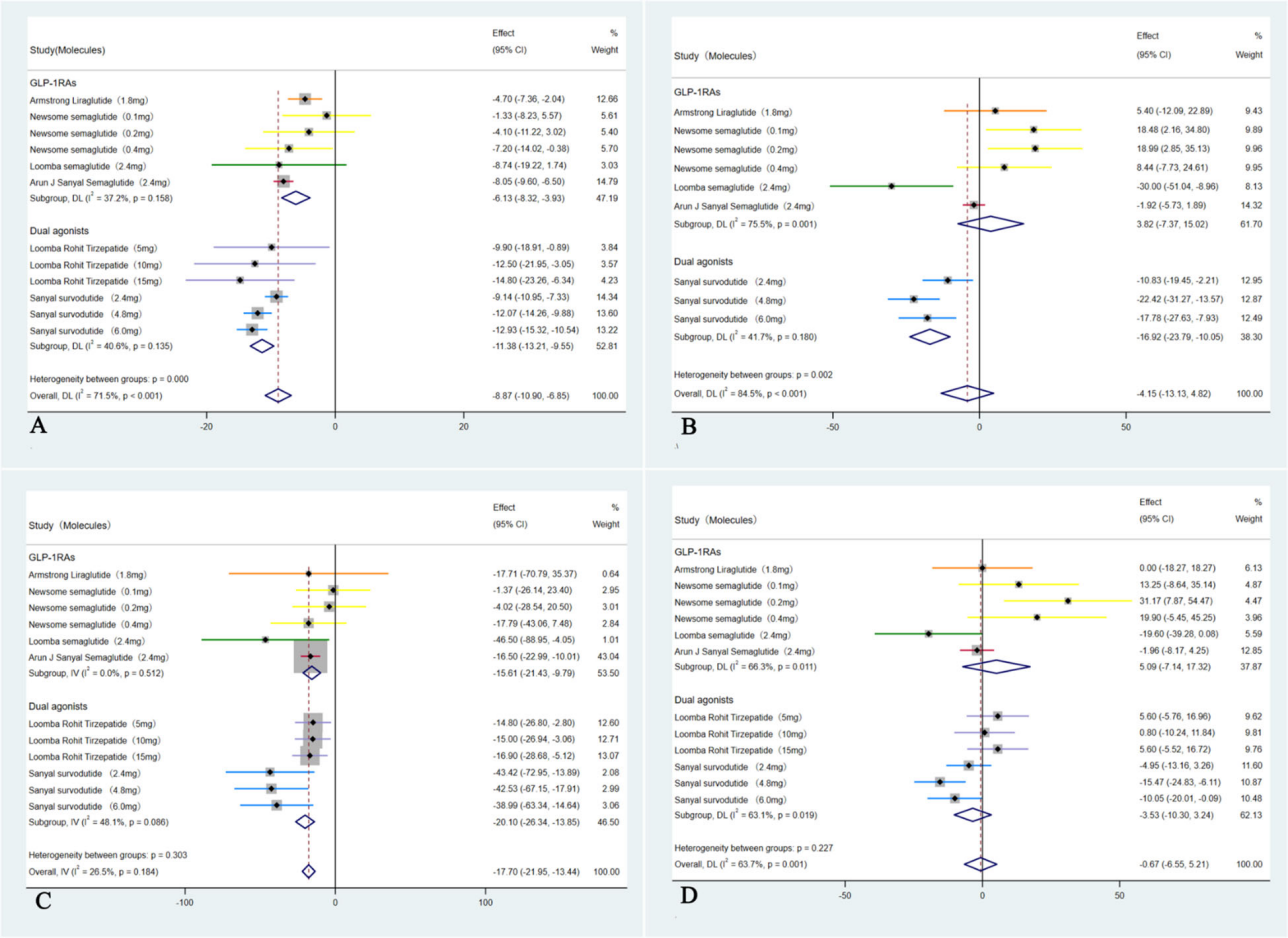
Forest plot of blood lipids levels in MASH patients treated with GLP-1RAs and dual agonists (**A**: body weight, **B**: TC, **C**: TG, **D**: LDL-C).

**Figure 6:**
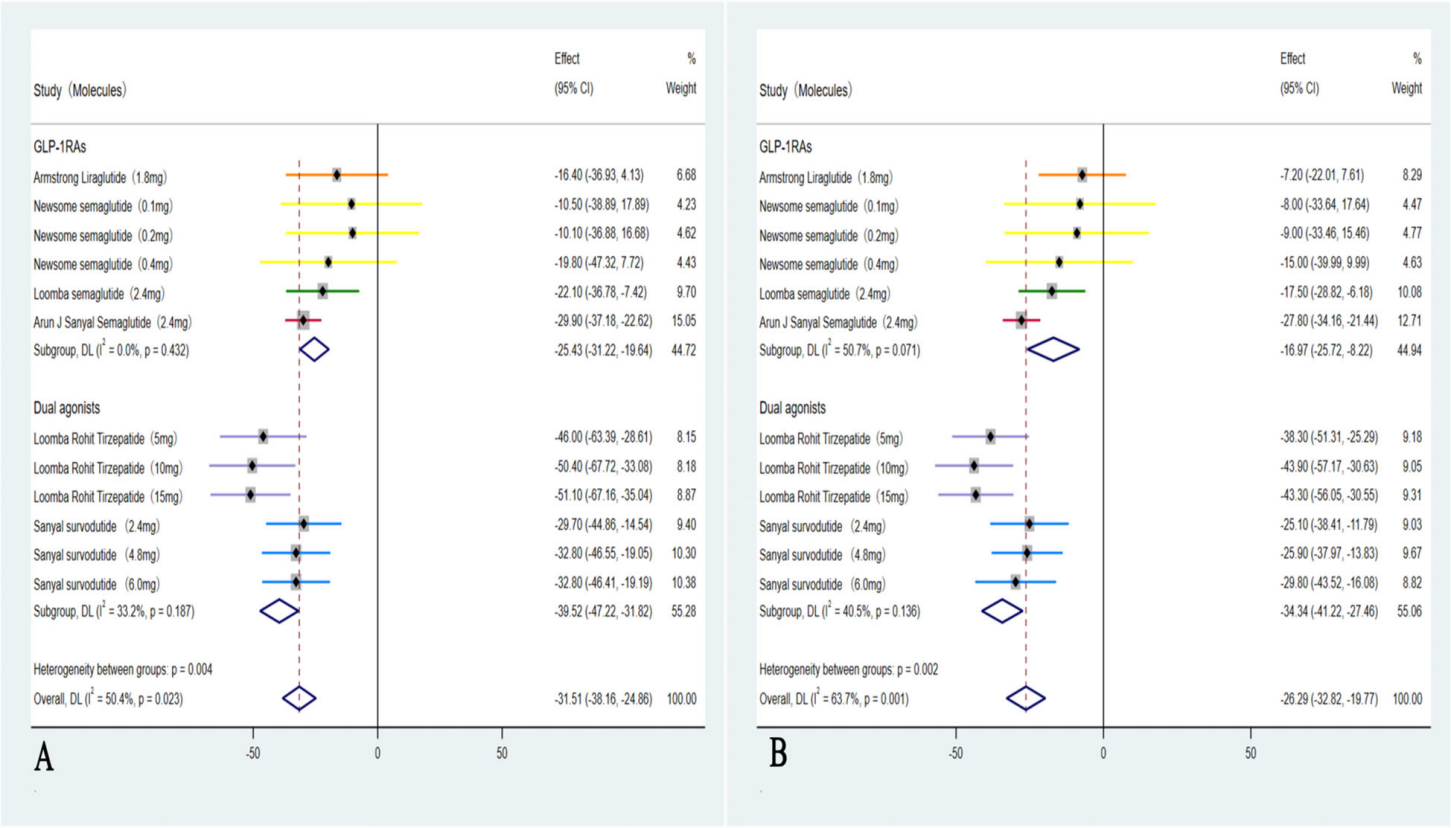
Forest plot of hepatic enzyme levels in MASH patients treated with GLP-1RAs and dual agonists (**A**: ALT, **B**: AST).

In these six studies (35–40), treatment with GLP-1RAs and dual agonists resulted in a significant pooled reduction in body weight compared to the placebo group, with a WMD of -8.87 kg (95% CI: -10.90 to -6.85 kg; I² = 71.5%, *p* < 0.01). In addition, GLP-1RAs and dual agonists demonstrated significant improvements in cardiovascular parameters, including TC (WMD = -4.15 mmol/L, 95% CI: -13.13 to 4.82, I² = 84.5%; *p* = 0.48) and TG (WMD = -17.70 mmol/L, 95% CI: -21.95 to -13.44, I² = 26.5%; *p* < 0.01). However, GLP-1RAs and dual agonists did not show a significant improvement in LDL-C levels (WMD = -0.67 mmol/L, 95% CI: -6.55 to 5.21, I² = 63.7%; *p* = 0.13) (**Figure 5**). GLP-1RAs and dual agonists also demonstrated significant improvements in hepatic enzyme levels, including ALT (WMD = -31.51 U/L, 95% CI: -38.16 to -24.86, I² = 50.4%; *p* < 0.01) and AST (WMD = -26.29 U/L, 95% CI: -32.82 to -19.77, I² = 63.7%; *p* < 0.01) (**Figure 6**).

#### 3.3.1 Subgroup analyses

**Figure 7:**
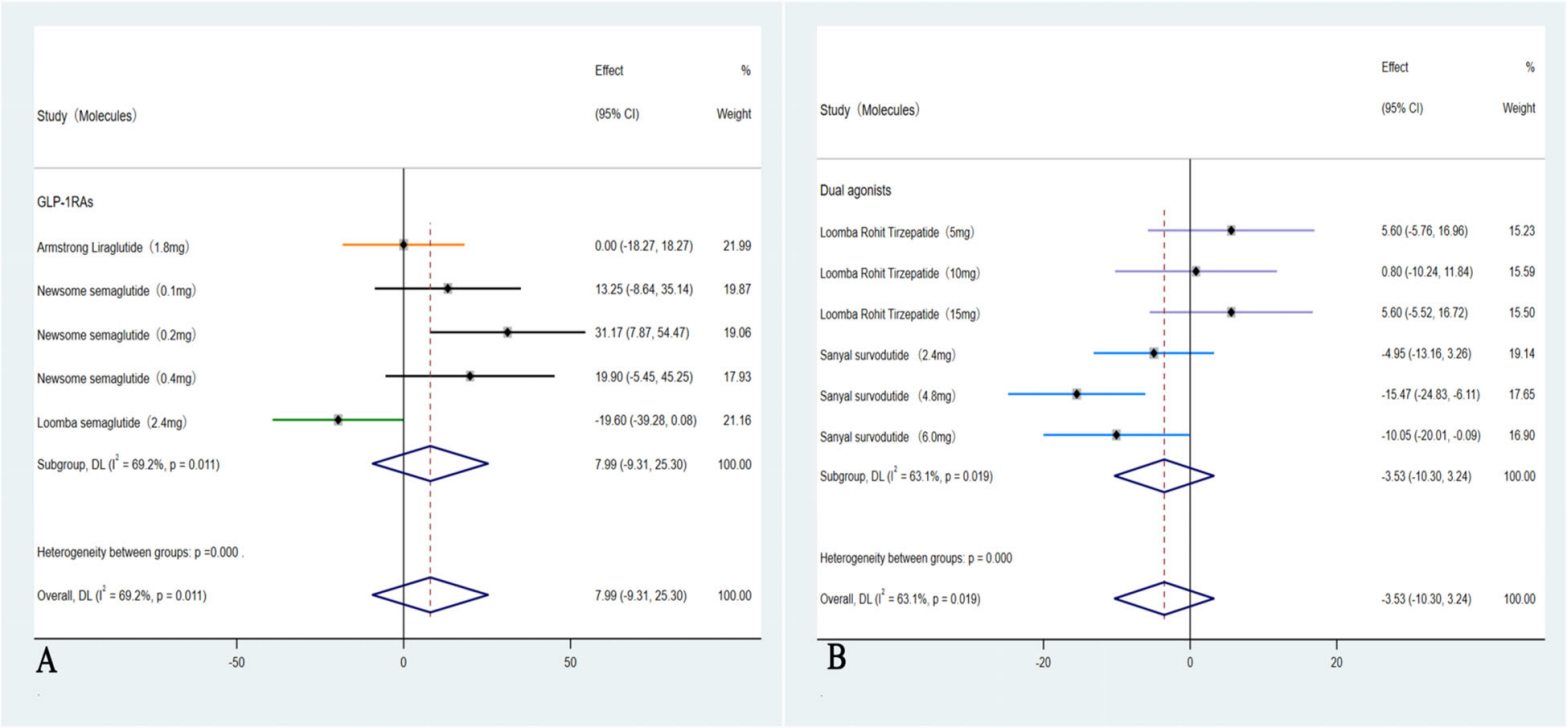
Forest plot of LDL-C changes in subgroups with <10% and ≥ 10% weight loss (**A**: <10%, **B**: ≥ 10%).

These five studies (35–39) were evaluated to further investigate the impact on LDL-C, in which a subgroup analysis revealed a significant reduction only among those with ≥10% weight loss group (Dual agonists), with an OR of –3.53 (95% CI: –10.30 to 3.24; I² = 63.1%; *p* = 0.04). In contrast, weight loss of <10% (GLP-1RAs) was not associated with a statistically significant improvement in LDL-C levels, with an OR of 7.99 (95% CI: –9.31 to 25.30; I² = 69.2%; *p* = 0.22) (**Figure 7**).

### 3.4 GI adverse events associated with GLP-1RAs and dual agonists

**Figure 8:**
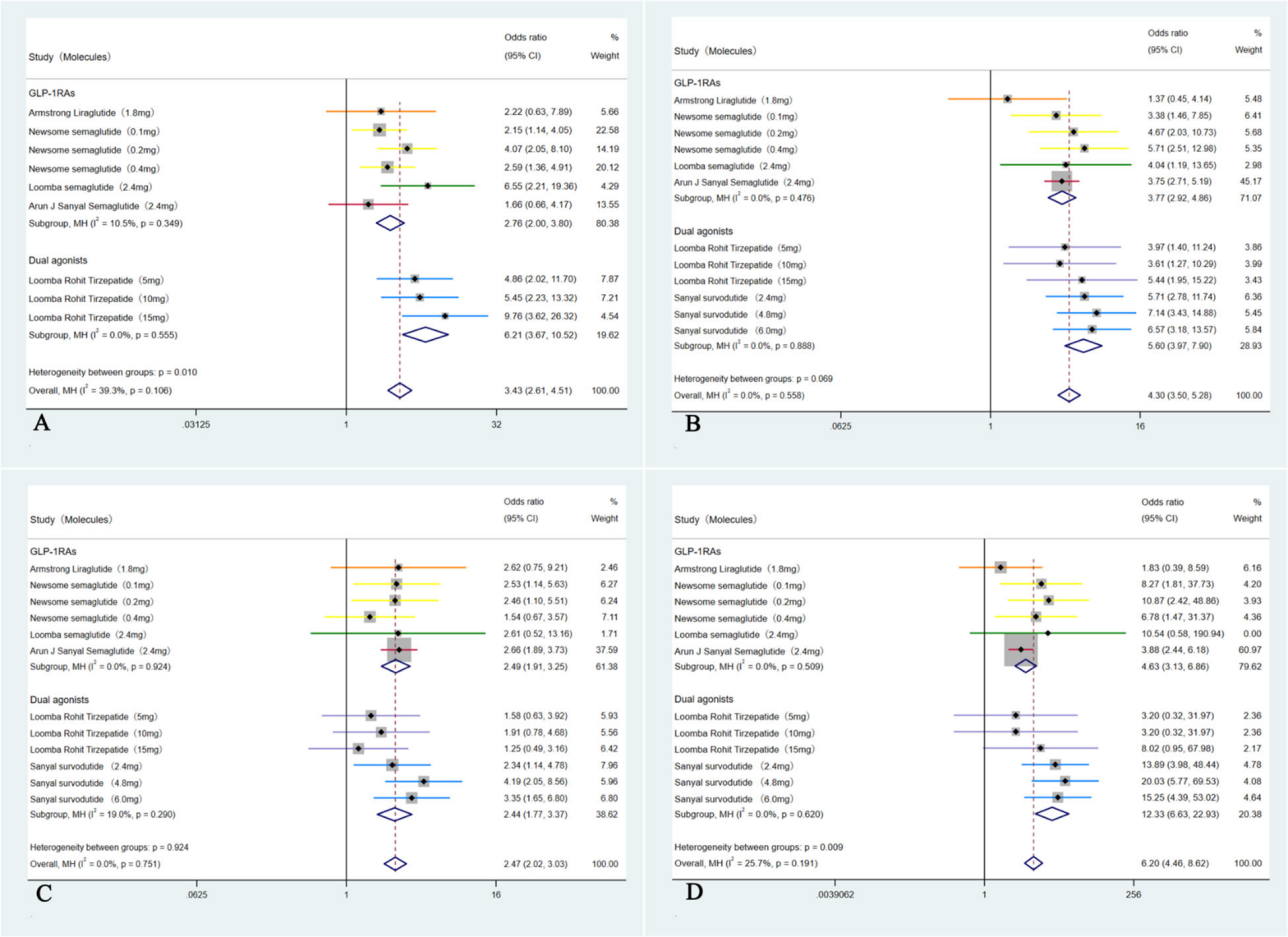
Forest plot of the GI adverse events in MASH patients treated with GLP-1RAs and dual agonists, with subgroups by GI events. (**A**: overall GI adverse events, **B**: nausea, **C**: diarrhea, **D**: vomiting).

In these six studies (35–40), patients treated with GLP-1RAs and dual agonists had significantly higher incidence rate of experiencing GI side effects compared to those receiving a placebo (28.31% (355/1254) VS 23.28% (193/829), OR = 3.43, 95% CI, 2.61 to 4.51, I² = 39.3%; *p* < 0.01). Among these adverse events, patients had a significantly higher incidence rate of experiencing nausea (41.28% (608/1473) VS 15.53% (162/1043), OR = 4.30, 95% CI, 3.50 to 5.28, I² = 0%; *p* < 0.01), followed by diarrhea (30.35% (447/1473) VS 16.49% (172/1043), OR = 2.47, 95% CI, 2.02 to 3.03, I² = 0%; *p* < 0.01) and vomiting (20.84% (307/1473) VS 3.06% (43/1403), OR = 6.20, 95% CI, 4.46 to 8.62, I² = 25.7%; *p* < 0.01) (**Figure 8**).

### 3.5 Sensitivity analysis

Sensitivity analysis using the leave-one-out method revealed substantial heterogeneity in the ORs for both MASH resolution and a ≥1-stage improvement in hepatic fibrosis (I² = 81.9%). The study by Sanyal et al. (39) was found to be the primary source of this heterogeneity. After excluding this study, heterogeneity decreased markedly (I² = 0%), while statistically significant outcomes in favor of GLP-1RAs and dual agonist therapy were maintained (OR = 2.65, 95% CI: 1.90 to 3.71, *p* < 0.01). Notably, despite variations in effect sizes and heterogeneity indices following the exclusion of individual studies, the pooled estimates remained statistically significant in all analyses. This consistency suggests that the results of the meta-analysis are robust.

## 4 Discussion

A total of 1,726 patients were included, and both GLP-1RAs and dual agonists demonstrated superior efficacy in hepatic fibrosis and resolution of MASH compared to placebo. They also had positive effects on weight loss, hepatic enzyme levels, and TC and TG levels. Notably, our analysis also showed that the degree of weight loss was a significant determinant of hepatic fibrosis improvement in MASH patients. Our analysis further demonstrated that treatment with GLP-1RAs and dual agonists had no substantial effect on LDL-C levels. Although GLP-1RAs and dual agonists do not directly lower LDL-C in patients with MASH, a significant reduction was observed in those who achieved substantial weight loss (≥10%). Conversely, weight loss of <10% was not associated with a significant decrease in LDL-C. Compared with placebo, GLP-1RAs and dual agonists significantly improved histological resolution of MASH without exacerbating hepatic fibrosis, and this finding was highly consistent across included studies. Even a modest weight loss (<10%) can positively impact MASH resolution, whereas greater weight loss (≥ 10%) is associated with a higher rate of MASH resolution and hepatic fibrosis improvement.

Hepatic fibrosis is a pathological response to chronic hepatocellular injury, characterized by excessive accumulation of ECM components (41). Without timely intervention, it can progress to cirrhosis, hepatic decompensation, or hepatocellular carcinoma, significantly increasing the risk of hepatic-related morbidity and mortality (41). Evidence indicates that patients with hepatic fibrosis have a 20% to 40% chance of developing hepatic-related complications, such as ascites and GI bleeding, within five years (42). Moreover, hepatic fibrosis is not only associated with hepatic-related complications but also independently increases the risk of cardiovascular mortality. This is due to the promotion of chronic systemic inflammation and metabolic dysregulation (43). Study shows that patients with moderate to severe hepatic fibrosis have a cardiovascular mortality rate 2 to 3 times higher than the general population. Among these patients, individuals with a Fibrosis 4 score (FIB-4) index greater than 3.25 face a 10-year cardiovascular mortality risk as high as 28.5% (44). In the early stages of hepatic fibrosis, the hepatic’s lobular structure remains intact, allowing activated hepatic stellate cells to revert to a quiescent state with appropriate intervention. Matrix metalloproteinases (MMPs) play a crucial role in degrading excess ECM, highlighting the reversible nature of early-stage fibrosis (45). Therefore, timely antifibrotic therapy not only improves hepatic-related outcomes but also mitigates cardiovascular risks by reducing systemic inflammation and metabolic disturbances. Reported reductions in cardiovascular event risks have been as high as 43% (46).

Excess body weight, particularly visceral adiposity, plays an important role in the pathogenesis of MASH by exacerbating hepatic lipid accumulation, oxidative stress, and inflammation, thereby accelerating the progression of hepatic fibrosis (47). Weight loss interventions, by reducing body weight, effectively decrease hepatic fat deposition, alleviating steatosis, the initial stage of MASH (10) (11). This weight loss-mediated effect not only mitigates lipotoxicity but also helps normalize hepatic function and reduces the production of pro-inflammatory cytokines, which are key mediators of hepatic fibrosis (48). Furthermore, the anti-inflammatory and anti-fibrotic effects of weight loss work synergistically with the direct hepatic actions of GLP-1RAs and dual agonists in activating hepatic tissue (49). Thus, modulating body weight through receptor activation represents a promising therapeutic approach, not only for promoting weight loss but also for reversing MASH and halting the progression of hepatic fibrosis.

Although GLP-1RAs and dual agonists have shown potential in improving MASH and hepatic fibrosis, the degree of weight loss plays a significant role in determining the effectiveness of these treatments. The analysis in this study revealed that treatment with GLP-1RAs and dual agonists led to significant improvements in hepatic fibrosis when weight loss was ≥ 10%. However, when weight loss was <10%, the improvement in hepatic fibrosis was not statistically significant. This suggests that the amelioration of hepatic fibrosis induced by GLP-1RAs and dual agonists may be an indirect outcome, dependent on the e degree of weight loss. Consequently, the degree of weight loss during GLP-1RA and dual agonist therapy could serve as a clinical marker to predict potential benefits for hepatic fibrosis. This finding underscores the limitation of relying solely on weight loss-mediated effects for fibrotic resolution. Therefore, there remains a significant need for direct anti-fibrotic therapies in MASH patients. This meta-analysis emphasized the distinct effects of GLP-1RAs and dual agonists on lipid metabolism in patients with MASH. The results demonstrate that GLP-1RAs and dual agonists significantly reduce TG and TC levels, but have a limited effect on LDL-C. The reduction in TG is primarily due to two mechanisms: inhibition of hepatic very Low Density Lipoprotein (VLDL) synthesis (50) and enhanced lipoprotein lipase (LPL) activity in adipose tissue (51). The decrease in TC is likely driven by reduced VLDL secretion, suppression of the Sterol Regulatory Element-Binding Protein-2 (SREBP-2) pathway (51), and synergistic metabolic effects, including improved insulin sensitivity and weight loss (52). The limited effect on LDL-C may be attributed to three factors: the absence of direct upregulation of (Low-Density Lipoprotein) LDL receptors, reduced VLDL to LDL conversion despite decreased VLDL production, and MASH related hepatic disturbances, such as downregulation of LDL receptors and impaired bile acid metabolism. These findings have important clinical implications. While GLP-1RAs and dual agonists effectively improve TG and TC metabolism in MASH patients, additional lipid-lowering strategies, such as statins, should be considered for those requiring comprehensive lipid management, particularly patients with elevated cardiovascular risk.

In addition, GLP-1RAs and dual agonists have demonstrated important hepatoprotective effects, including notable reductions in serum AST and ALT levels (53). As typical markers of hepatocellular injury and inflammation, elevated ALT and AST levels directly reflect liver cell damage (54). The studies suggest a dual mechanism of action: firstly, these agents exert direct hepatoprotective effects by reducing hepatic lipid accumulation and suppressing inflammatory responses (55); secondly, they confer beneficial effects on hepatic fibrosis (56, 57).This multi-targeted mechanism provides a strong theoretical rationale for applying GLP-1RAs and dual agonists in the treatment of MASH.

This study found that the use of GLP-1RAs and dual agonists is associated with adverse effects, including nausea, vomiting, and diarrhea, with nausea being the most frequently reported. Among the six included studies, the overall incidence of serious adverse events (SAEs) was 12%. Among GLP-1RAs, in the liraglutide treatment group (35), the incidence of SAEs was 8%. GI adverse events, including nausea (46%), diarrhea (38%), and vomiting (19%), primarily occurred during the initial 1–4 weeks of treatment, with nausea peaking in weeks 1–2 (>38%). The treatment discontinuation rate due to adverse events was 8%, including two cases discontinued because of nausea. In the semaglutide treatment group (36), the incidence of serious SAEs was 16%. GI adverse events, including nausea (36%), diarrhea (25%), and vomiting (17%), primarily occurred during the dose-escalation period (weeks 1–16). Treatment discontinuation due to adverse events was 7%, with 4% attributable to GI events. In another semaglutide study (37), the incidence of serious SAEs was 13%. GI events, including nausea (45%), diarrhea (19%), and vomiting (17%), primarily occurred during the dose-escalation period (weeks 1–16). The treatment discontinuation rate due to adverse events was 6%, with two cases due to nausea and one due to vitreous detachment. In the semaglutide treatment group (40), the incidence of serious SAEs was 13.4%. GI adverse events, including nausea (36%), vomiting (19%), and diarrhea (27%), were primarily reported during the dose-escalation phase (weeks 1–24). The treatment discontinuation rate due to adverse events was 2.6%. Among dual receptor agonists, in the tirzepatide treatment group (38), the incidence of serious SAEs was 6%, comparable to the placebo group, with no significant difference observed. GI adverse events, including nausea (38%), diarrhea (32%), and vomiting (9%), were most frequently reported during the dose-escalation phase (weeks 1–12). The treatment discontinuation rate due to adverse events was 4%. In the survodutide treatment group (39), the incidence of serious SAEs was 8%. GI adverse events were more frequent, including nausea (66%), diarrhea (49%), and vomiting (41%), primarily occurring during the rapid dose-escalation phase (weeks 1–24). These events led to treatment discontinuation in 20% of patients, of which 16% were attributable to GI events. Overall, GI reactions are the most common adverse events associated with these drugs, with average incidences of nausea, diarrhea, and vomiting of 41.28%, 30.35%, and 20.84%, respectively. Other common adverse events include hypoglycemia, occurring more frequently in patients with diabetes (3.4%–34%) than in non-diabetic individuals (0.3%–0.6%), and decreased appetite (13%–31%). SAEs, such as malignancies or cardiovascular events, have been reported occasionally, but are generally not directly attributable to the drugs.

The high incidence of these GI adverse events represents a major limitation to the widespread use of GLP-1RAs and dual agonists (58–60).Consequently, discontinuation rates across GLP-1RA- and dual agonist-based MASH therapies show substantial heterogeneity, primarily driven by their mechanisms of action, including delayed gastric emptying and altered GI motility (61, 62).

In addition, several studies have shown that nausea and vomiting are more common during the first 4–5 weeks of treatment, typically resolving within 8 days after symptom onset (63, 64). Diarrhea typically occurs during the initial 2–4 weeks of treatment. To minimize these GI side effects, clinical guidelines recommend initiating treatment at a low dose and gradually titrating to the therapeutic dose (63). Beyond GI side effects, GLP-1RAs and dual agonists have also been associated with adverse events affecting the pancreas, biliary, and nervous system (65), as well as a 27% incidence of cholelithiasis and a 36% incidence of cholecystitis (66). Neurological adverse effects primarily involve central nervous system symptoms, such as headache, with an incidence of 5–15% (67). Although these adverse events are generally rare (<5%), individual responses may vary significantly, influenced by factors such as comorbidities, baseline disease status, and treatment regimens (67). Therefore, a thorough pre-treatment risk assessment, considering factors such as obesity, weight loss history, pancreatic diseases, neurological disorders, and biliary diseases, is essential. Furthermore, regular monitoring every 12 weeks, including measurements of serum amylase and lipase, is recommended to facilitate early detection of adverse events (68).

## 5 Limitations

This study systematically analyzed six qualified RCTs published in The New England Journal of Medicine and The Lancet, which evaluated the therapeutic effects of GLP-1 RAs and their dual agonists in patients with MASH. Given the relatively recent clinical introduction of these agents and the strict inclusion criteria requiring hepatic biopsy for MASH diagnosis, the limited number of eligible studies may constrain the generalizability and clinical applicability of the findings. Furthermore, substantial variations in baseline characteristics, such as age, gender distribution, duration of intervention, and comorbidities, were observed across the studies. Although statistical methods were employed to control for these confounding factors, the potential impact of these differences on the final results remains considerable. In addition, discrepancies in the types of GLP-1RAs and dual agonists used and the duration of treatment further complicate comparisons of drug efficacy and limit the accurate assessment of long-term effectiveness and safety.

## 6 Conclusion

This meta-analysis demonstrates that GLP-1RAs and dual agonists can effectively improve hepatocellular inflammation and hepatic fibrosis in patients with MASH. Specifically, when patients achieve a weight loss of ≥10%, these drugs are associated with significant improvements in hepatic fibrosis, whereas the effect is limited in those with less than 10% weight loss. In terms of lipid management, they effectively reduce TC and TG levels. However, GLP-1RAs and dual agonists do not directly lower LDL-C in patients with MASH; a significant reduction was observed only in those who achieved substantial weight loss (≥10%), whereas weight loss of <10% was not associated with a significant decrease in LDL-C. This finding suggests that for patients requiring comprehensive cardiovascular risk management, additional lipid-lowering strategies may be needed to enhance the intervention. From the perspective of hepatoprotective mechanisms, these drugs may play a dual role by reducing hepatic steatosis and suppressing inflammatory cascade responses through lowering serum AST and ALT levels. Based on these findings, when using GLP-1RAs and dual agonists clinically to treat hepatic fibrosis, the impact of weight loss should be carefully considered, and close monitoring of LDL-C levels is recommended to reduce cardiovascular risk. In addition, the common GI side effects (e.g., nausea) associated with these therapies highlight the need for gradual dose titration and routine monitoring. Further studies are warranted to explore the long-term hepatic and cardiovascular outcomes of these therapies in patients with MASH.

## Data Availability

The original contributions presented in the study are included in the article/**Supplementary Material**. Further inquiries can be directed to the corresponding author.
