# Supplementary material for: Comparative effectiveness of GLP-1 receptor agonists and dual agonists in the treatment of patients with metabolic dysfunction associated steatohepatitis: a systematic review and meta-analysis
Source: Front Endocrinol (Lausanne). 2025 Oct 8;16:1681965. doi: 10.3389/fendo.2025.1681965 (PMC12540095; doi:10.3389/fendo.2025.1681965)
Supplement: Supplementary file 1 [file SupplementaryFile1.docx]

**SUPPLEMENTARY MATERIAL LEGEND**

**Supplemental Materials 1**. **Eligibility Criteria**

| **Criteria** | **Inclusion** | **Exclusion** |
| --- | --- | --- |
| Conditions | Studies reporting on the resolution of hepatic fibrosis and MASH (Metabolic Dysfunction-Associated Steatohepatitis), as well as secondary outcomes. | Studies with inconsistent research designs, or those lacking or having insufficient data to extract key information (such as outcome measures, effect sizes, and sample sizes). |
| Population | Participants must have a confirmed diagnosis of MASH through hepatic biopsy. |  |
| Study Design | Randomized controlled trials | Non - randomized studies  Animal studies |
| Outcome Measurement | Primary Endpoint（by hepatic biopsy）   1. Resolution of MASH without worsening of hepatic fibrosis 2. An improvement with ≥1-stage hepatic fibrosis without worsening of MASH 3. Contributed to MASH resolution accompanied by ≥1-stage improvement in hepatic fibrosis   Secondary Endpoints   1. Changes in body weight 2. Changes in total cholesterol 3. Changes in triglycerides 4. Changes in low Density Lipoprotein 5. Changes in serum hepatic enzyme levels 6. Alanine aminotransferase (ALT) 7. Aspartate aminotransferase (AST) 8. Gastrointestinal adverse events 9. Subgroup analyses were carried out by categorizing weight loss into <10% (GLP - 1RAs) and ≥10% (Dual agonists) to further evaluate how weight loss impacts primary outcomes. 10. A further subgroup analysis was performed to examine the impact of weight loss (<10% vs. ≥ 10%) on LDL-c levels. |  |
| Publication Type | • Published primary research  • Unpublished thesis | Abstract only  Conferences  Letters  Editorials  Editorial comments  Short reports  Duplicate articles  Oral communications  Book chapter reviews |
| Language | English |  |
| Year of Publication | No limit |  |

**Supplemental Materials 2. Index Terms and Keywords used in the seven databases**

**Database: Scopus**

| **Search No.** | **Indexed and Keyword Terms** |
| --- | --- |
| #1 | ( TITLE-ABS-KEY ( "Non-alcoholic Fatty hepatic Disease" ) OR TITLE-ABS-KEY ( mash ) OR TITLE-ABS-KEY ( "Fatty hepatic, Nonalcoholic*" ) OR TITLE-ABS-KEY ( nafld ) OR TITLE-ABS-KEY ( "Nonalcoholic Steatohepatitis" ) OR TITLE-ABS-KEY ( " hepatics, Nonalcoholic Fatty*" ) OR TITLE-ABS-KEY ( "Non-alcoholic Fatty hepatic Disease*" ) AND TITLE-ABS-KEY ( fibrotic ) OR TITLE-ABS-KEY ( fibrosis* ) OR TITLE-ABS-KEY ( cirrhosis* ) ) |
| #2 | ( TITLE-ABS-KEY ( "Glucagon-Like Peptide-1 Receptor Agonists" ) OR TITLE-ABS-KEY ( "GLP-1 Agonists" ) OR TITLE-ABS-KEY ( "Agonists, GLP-1" ) OR TITLE-ABS-KEY ( "GLP-1 Analogs*" ) OR TITLE-ABS-KEY ( "GLP1R Agonists*" ) OR TITLE-ABS-KEY ( "GLP 1 Receptor Agonists*" ) OR TITLE-ABS-KEY ( "Agonists, GLP1*" ) OR TITLE-ABS-KEY ( "semaglutide" ) OR TITLE-ABS-KEY ( "dulaglutide" ) OR TITLE-ABS-KEY ( "survodutide" ) OR TITLE-ABS-KEY ( "Tirzepatide" ) ) |
| #3 | (TITLE-ABS-KEY (“GIP receptor”) OR TITLE-ABS-KEY (“glucose-dependent insulinotropic polypeptide receptor”) OR TITLE-ABS-KEY (“receptor, gastric inhibitory polypeptide”)) |
| #4 | (TITLE-ABS-KEY (“Receptors, Gastrointestinal Peptides”) OR TITLE-ABS-KEY (“Gastrointestinal Peptides Receptors”) OR TITLE-ABS-KEY (“Intestinal Hormone Receptors”) OR TITLE-ABS-KEY (“Gastrointestinal Hormone Receptors”) OR TITLE-ABS-KEY (“Hormone Receptors, Intestinal”)) |
| #5 | (TITLE-ABS-KEY (“Receptor, Glucagon”) OR TITLE-ABS-KEY (“Glucagon Receptor*”)) |
| #6 | (TITLE-ABS-KEY (“Losses, Weight”) OR TITLE-ABS-KEY (“Weight Reduction*”) OR TITLE-ABS-KEY (“body mass index”) OR TITLE-ABS-KEY (bmi)) |
| #7 | #2 OR #3 OR #4 OR #5 OR #6 |
| #8 | #1 AND #7 |

**Database: EMBASE**

| **Search No.** | **Indexed and Keyword Terms** |
| --- | --- |
| #1 | 'non-alcoholic fatty hepatic disease'/exp |
| #2 | 'Nonalcoholic fatty hepatic disease*’: ab, ti OR 'NAFLD’: ab, ti OR 'fatty hepatic, nonalcoholic*': ab, ti OR NAFLD: ab, ti OR 'nonalcoholic steatohepatitis’: ab, ti OR ' hepatics, nonalcoholic fatty*’: ab, ti OR 'MASH’: ab, ti OR 'non-alcoholic fatty hepatic disease*’: ab, ti |
| #3 | #1 OR #2 |
| #4 | 'Glucagon-Like Peptide-1 Receptor Agonists'/exp OR 'Gastric Inhibitory polypeptide Receptor'/exp OR 'Receptors, Gastrointestinal Hormone*'/exp OR 'Receptors, Glucagon'/exp OR 'Glucagon Gastric Inhibitory Polypeptide'/exp OR 'Weight Loss'/exp |
| #5 | 'Glucagon Like Peptide 1 Receptor Agonists’: ti, ab OR 'GLP-1 Agonists’: ti, ab OR "Agonists, GLP-1’: ti, ab OR 'GLP-1 Analogs*’: ti, ab OR 'GLP1R Agonists*’: ti, ab OR 'GLP 1 Receptor Agonists*': ti, ab OR "Agonists, GLP1*’: ti, ab |
| #6 | 'GIP receptor’: ti, ab OR 'glucose-dependent insulinotropic polypeptide receptor’: ti, ab OR 'receptor, gastric inhibitory polypeptide’: ti, ab |
| #7 | 'Receptors, Gastrointestinal Peptides’: ti, ab OR 'Gastrointestinal Peptides Receptors’: ti, ab OR 'Intestinal Hormone Receptors’: ti, ab OR 'Hormone Receptors, Intestinal’: ti, ab OR 'Gastrointestinal Hormone Receptors’: ti, ab |
| #8 | 'Receptor, Glucagon’: ti, ab OR 'Glucagon Receptor*’: ti, ab |
| #9 | 'Losses, Weight’: ti, ab OR 'Weight Reduction*’: ti, ab OR "body mass index’: ti, ab OR BMI: ti, ab |
| #10 | 'semaglutide’: ti, ab OR 'dulaglutide’: ti, ab OR 'survodutide’: ti, ab OR 'Tirzepatide’: ti, ab |
| #11 | #4 OR #5 OR #6 OR #7 OR #8 OR #9 OR #10 |
| #12 | #3 AND #11 |

**Database: PubMed**

| **Search No.** | **Indexed and Keyword Terms** |
| --- | --- |
| #1 | "Non-alcoholic Fatty hepatic Disease"[Mesh] |
| #2 | (((((((" Nonalcoholic Fatty hepatic Disease*"[Title/Abstract]) OR ("Fatty hepatic, Nonalcoholic*"[Title/Abstract])) OR (NAFLD[Title/Abstract] OR "Nonalcoholic Steatohepatitis"[Title/Abstract])) OR (" hepatics, Nonalcoholic Fatty*"[Title/Abstract])) OR ("Non-alcoholic Fatty hepatic Disease*"[Title/Abstract])) AND (fibrotic[Title/Abstract])) OR (Fibrosis*[Title/Abstract])) OR (Cirrhosis*[Title/Abstract]) |
| #3 | #1 OR #2 |
| #4 | ((((("Glucagon-Like Peptide-1 Receptor Agonists"[Mesh Terms]) OR ("Gastric Inhibitory polypeptide Receptor"[Mesh Terms])) OR ("Receptors, Gastrointestinal Hormone*"[Mesh Terms])) OR ("Receptors, Glucagon"[Mesh Terms])) OR ("Glucagon Gastric Inhibitory Polypeptide"[Mesh Terms])) OR ("Weight Loss"[Mesh Terms]) |
| #5 | (((((("Glucagon Like Peptide 1 Receptor Agonists"[Title/Abstract]) OR ("GLP-1 Agonists"[Title/Abstract])) OR ("Agonists, GLP-1"[Title/Abstract])) OR ("GLP-1 Analogs*"[Title/Abstract])) OR ("GLP1R Agonists*"[Title/Abstract])) OR ("GLP 1 Receptor Agonists*"[Title/Abstract])) OR ("Agonists, GLP1*"[Title/Abstract]) |
| #6 | (("GIP receptor"[Title/Abstract]) OR ("glucose-dependent insulinotropic polypeptide receptor"[Title/Abstract])) OR ("receptor, gastric inhibitory polypeptide"[Title/Abstract]) |
| #7 | (((("Receptors, Gastrointestinal Peptides"[Title/Abstract]) OR ("Gastrointestinal Peptides Receptors"[Title/Abstract])) OR ("Intestinal Hormone Receptors"[Title/Abstract])) OR ("Hormone Receptors, Intestinal"[Title/Abstract])) OR ("Gastrointestinal Hormone Receptors"[Title/Abstract]) |
| #8 | ("Receptor, Glucagon"[Title/Abstract]) OR ("Glucagon Receptor*"[Title/Abstract]) |
| #9 | ((("Losses, Weight"[Title/Abstract]) OR ("Weight Reduction*"[Title/Abstract])) AND ("body mass index"[Title/Abstract])) OR (BMI[Title/Abstract]) |
| #10 | "semaglutide"[Title/Abstract] OR "dulaglutide"[Title/Abstract] OR "survodutide"[Title/Abstract] OR "Tirzepatide"[Title/Abstract] |
| #11 | #4 OR #5 OR #6 OR #7 OR #8 OR #9 OR #10 |
| #12 | #3 AND #11 |

**Database: Cochrane**

| **Search No.** | **Indexed and Keyword Terms** |
| --- | --- |
| #1 | Mesh descriptor: [Non-alcoholic Fatty hepatic Disease] explode all trees |
| #2 | (Fatty hepatic, Nonalcoholic): ti, ab OR (Fatty hepatics, Nonalcoholic): ti, ab, kw OR (hepatic, Nonalcoholic Fatty): ti, ab, kw OR (hepatics, Nonalcoholic Fatty): ti, ab, kw OR (Nonalcoholic Fatty hepatic): ti, ab, kw |
| #3 | #1 OR #2 |
| #4 | Mesh descriptor: [Glucagon-Like Peptide-1 Receptor Agonists] explode all trees |
| #5 | (GLP-1 Receptor Agonists): ti, ab, kw OR (GLP 1 Receptor Agonists): ti, ab, kw OR (Incretin Mimetics): ti, ab, kw OR (GLP-1 Analogs): ti, ab, kw OR (GLP 1 Analogs): ti, ab, kw |
| #6 | #4 OR #5 |
| #7 | Mesh descriptor: [Gastric Inhibitory Polypeptide] explode all trees |
| #8 | #8 (Inhibitory Polypeptide, Gastric): ti, ab, kw OR (Gastric-Inhibitory Polypeptide): ti, ab, kw OR (Polypeptide, Gastric-Inhibitory): ti, ab, kw OR (Glucose Dependent Insulinotropic Peptide): ti, ab, kw OR (Glucose Dependent Insulinotropic Peptide): ti, ab, kw |
| #9 | (Insulinotropic Peptide, Glucose-Dependent): ti, ab, kw OR (Peptide, Glucose-Dependent Insulinotropic): ti, ab, kw OR (Peptide, Glucose-Dependent Insulinotropic): ti, ab, kw OR (Peptide, Glucose-Dependent Insulin-Releasing): ti, ab, kw OR (Insulin-Releasing Peptide, Glucose-Dependent): ti, ab, kw |
| #10 | #7 OR #8 OR #9 |
| #11 | Mesh descriptor: [Receptors, Glucagon] explode all trees |
| #12 | (Glucagon Receptors): ti, ab, kw OR (Receptor, Glucagon): ti, ab, kw OR (Receptors, Glucagon): ti, ab, kw |
| #13 | #11 OR #12 |
| #14 | #6 OR #10 OR #13 |
| #15 | Semaglutide OR Liraglutide OR Exenatide OR Dulaglutide |
| #16 | #14 OR #15 |
| #17 | #3 AND #16 |

**Database: Web of Science**

| **Search No.** | **Indexed and Keyword Terms** |
| --- | --- |
| 1 | TS= (((((((((("Non-alcoholic Fatty hepatic Disease")) OR ("Fatty hepatic, Nonalcoholic*")) OR (NAFLD)) OR TS=(MASH)) OR ("Nonalcoholic Steatohepatitis")) OR (" hepatics, Nonalcoholic Fatty*")) OR ("Non-alcoholic Fatty hepatic Disease*")) AND (fibrotic)) OR (Fibrosis*)) OR (Cirrhosis*) |
| 2 | TS= ((((((("Glucagon-Like Peptide-1 Receptor Agonists")) OR ("GLP-1 Agonists")) OR ("Agonists, GLP-1")) OR ("GLP-1 Analogs*")) OR ("GLP1R Agonists*")) OR ("GLP 1 Receptor Agonists*")) OR ("Agonists, GLP1*") |
| 3 | TS= (((("Gastric Inhibitory polypeptide Receptor")) OR ("GIP receptor")) OR ("glucose-dependent insulinotropic polypeptide receptor")) OR ("receptor, gastric inhibitory polypeptide") |
| 4 | TS= ((((("Glucagon Gastric Inhibitory Polypeptide")) OR ("Gastrointestinal Peptides Receptors")) OR ("Intestinal Hormone Receptors")) OR ("Hormone Receptors, Intestinal")) OR ("Gastrointestinal Hormone Receptors") |
| 5 | TS= ((((("Weight Loss")) OR ("Losses, Weight")) OR ("Weight Reduction*")) AND ("body mass index")) OR (BMI) |
| 6 | #2 OR #3 OR #4 OR #5 |
| 7 | #1 AND #6 |

**Supplemental Materials 3. Full details of risk of bias assessments**

**Armstrong 2016**

| Domain | Judgement | Support for judgement |
| --- | --- | --- |
| Random sequence generation (selection bias) | Low risk | “Patients were randomly assigned (1:1) using a computer-generated, centrally administered procedure, stratified by trial center and diabetes status.” |
| Allocation concealment (selection bias) | Low risk | “Allocation concealment was achieved by packaging both liraglutide and placebo groups with a unique identification number” “A master control list... was retained at the trials unit and was accessible only by the database programmer and the statistician.” |
| Blinding of participants and personnel (performance bias) | Low risk | “Patients, investigators, clinical trial site staff, and pathologists were masked to treatment assignment throughout the study. |
| Blinding of outcome assessment (detection bias) | Low risk | “Two independent hepatic histopathologists... were blinded to study treatment allocation and clinical or laboratory information.” |
| Incomplete outcome data (attrition bias) | Low risk | “Three patients in each treatment group missed the end-of-treatment biopsies and withdrew from treatment.” |
| Selective reporting (reporting bias) | Low risk | The study comprehensively reported the predefined primary and secondary outcomes (e.g., histological improvement, metabolic parameters), with no evidence of selective reporting. |
| Other bias | Low risk | "Although the study was partially funded by Novo Nordisk (a pharmaceutical manufacturer), the authors declared conflicts of interest and emphasized that the funder had no involvement in the study design or data analysis. No other significant biases were identified." |
| Overall Assessment | Low risk | Low risk of bias in all domains |

**Newsome 2021**

| Domain | Judgement | Support for judgement |
| --- | --- | --- |
| Random sequence generation (selection bias) | Unclear risk | Patients were randomly assigned, in a 3:3:3:1:1:1 ratio, to receive once-daily subcutaneous semaglutide at a dose of 0.1, 0.2, or 0.4 mg or corresponding placebo.” but did not elaborate on the specific randomization methodology. |
| Allocation concealment (selection bias) | Low risk | “Randomization was performed with the use of an interactive Web-response system and stratified according to geographic region, type 2 diabetes status, and baseline fibrosis stage.” |
| Blinding of participants and personnel (performance bias) | Low risk | “a double-blind phase 2 trial” “blinding of treatment assignments within dose levels” |
| Blinding of outcome assessment (detection bias) | Low risk | “In total, 320 patients... 302 patients (94%) completed the trial... and 285 patients (89%) completed treatment.” “Missing data were alternatively handled by multiple imputation from the placebo group” Missing data existed but were appropriately addressed, suggesting a low risk of attrition bias. |
| Incomplete outcome data (attrition bias) | Low risk | Primary and secondary endpoints were fully reported in the main text, while the complete list of endpoints was provided in supplementary materials. The absence of apparent selective reporting suggests minimal risk of reporting bias. |
| Selective reporting (reporting bias) | Low risk | The trial was funded by the pharmaceutical company Novo Nordisk, which may present a potential conflict of interest. Additionally, the sample size was adjusted during the trial (based on placebo response data from other studies), a modification that could introduce bias. |
| Other bias | NA | NA |
| Overall Assessment | Some  concern | Some concern for risk of bias in one domain. |

**Loomba 2023**

| Domain | Judgement | Support for judgement |
| --- | --- | --- |
| Random sequence generation (selection bias) | Unclear risk | patients were randomly assigned (2:1) via an interactive web response system, stratified by presence or absence of type 2 diabetes” but does not specify the method for generating the random sequence. |
| Allocation concealment (selection bias) | Unclear risk | “Randomization was done centrally using an interactive web response system and stratified for presence or absence of type 2 diabetes. Calyx (formerly Parexel) generated the randomization list.” The literature mentions that allocation was conducted via an interactive web response system, but does not explicitly state whether the allocation sequence was concealed from researchers and participants. |
| Blinding of participants and personnel (performance bias) | Low risk | “double-blind, placebo-controlled” |
| Blinding of outcome assessment (detection bias) | Unclear risk | “Pathologist evaluation included presence or absence of NASH, fibrosis stage... but not time” Although the pathologists were unaware of the patient characteristics and treatment allocation, it was not explicitly stated whether blinding of outcome assessors was implemented. |
| Incomplete outcome data (attrition bias) | Low risk | The literature states that 90% of patients completed the treatment, and an intention-to-treat (ITT) analysis was employed, with missing data imputed as non-response. |
| Selective reporting (reporting bias) | Low risk | The study reported all pre-specified primary and secondary outcomes, including any changes to the primary endpoint and the reasons for these modifications. |
| Overall Assessment | Some  concern | Some concern for risk of bias in one domain. |

**Loomba 2024**

| Domain | Judgement | Support for judgement |
| --- | --- | --- |
| Random sequence generation (selection bias) | Unclear risk | patients were randomly assigned (2:1) via an interactive web response system, stratified by presence or absence of type 2 diabetes” but does not specify the method for generating the random sequence (such as computer-generated, random number tables. |
| Allocation concealment (selection bias) | Low risk | The literature mentions that allocation was conducted via an interactive web response system, but does not explicitly state whether the allocation sequence was concealed from researchers and participants. |
| Blinding of participants and personnel (performance bias) | Low risk | “double-blind, placebo-controlled”，The trial utilized a visually matched placebo to ensure blinding of participants and investigators. |
| Blinding of outcome assessment (detection bias) | Unclear risk | “Pathologist evaluation included presence or absence of NASH, fibrosis stage... but not time” Although the pathologists were unaware of the patient characteristics and treatment allocation, it was not explicitly stated whether blinding of outcome assessors was implemented. |
| Incomplete outcome data (attrition bias) | Low risk | The literature states that 90% of patients completed the treatment, and an intention-to-treat (ITT) analysis was employed, with missing data imputed as non-response. |
| Selective reporting (reporting bias) | Low risk | The study reported all pre-specified primary and secondary outcomes, including any changes to the primary endpoint and the reasons for these modifications. |
| Other bias | NA | NA |
| Overall Assessment | Some  concern | Some concern for risk of bias in one domain. |

**Sanyal 2024**

| Domain | Judgement | Support for judgement |
| --- | --- | --- |
| Random sequence generation (selection bias) | Low risk | "Randomization lists were generated with the use of a pseudorandom number generator" |
| Allocation concealment (selection bias) | Unclear risk | The article only mentions the generation of the randomization list but does not specify how the unpredictability of the allocation process was ensured, resulting in unclear risks of selection bias. |
| Blinding of participants and personnel (performance bias) | Low risk | "Participants, investigators, safety central reviewers, and personnel involved in trial conduct or analysis... were unaware of the trial-group assignments" |
| Blinding of outcome assessment (detection bias) | Low risk | "Baseline and end-of-treatment values were derived from one central pathologist... who was unaware of trial-group assignments" |
| Incomplete outcome data (attrition bias) | Low risk | "Modified intention-to-treat population... missing data imputed as nonresponse""95.9% completed the trial" The study employed a modified intention-to-treat (Mitt) analysis, with a low dropout rate (4.1%), and baseline characteristics were well-balanced across groups. |
| Selective reporting (reporting bias) | Low risk | "The trial protocol was pre-registered (NCT04771273)""All predefined endpoints were reported (Table S1)" |
| Other bias | NA | NA |
| Overall Assessment | Some  concern | Some concern for risk of bias in one domain. |

**Arun J Sanyal 2025**

| Domain | Judgement | Support for judgement |
| --- | --- | --- |
| Random sequence generation (selection bias) | Low risk | The trial is explicitly described as a "multicenter, randomized, double-blind, placebo-controlled trial," and it mentions that "patients were randomly assigned in a 2:1 ratio by means of a centralized system." |
| Allocation concealment (selection bias) | Low risk | The trial used a "centralized system" for random allocation, and semaglutide and placebo were provided in "prefilled pen injectors" with identical appearance and administration methods. |
| Blinding of participants and personnel (performance bias) | Low risk | The trial is clearly labeled as "double-blind," and semaglutide and placebo are provided in "prefilled pen injectors" with the same appearance. The administration route (weekly subcutaneous injection) and dose escalation protocol (gradually increasing from 0.25 mg to 2.4 mg) are implemented uniformly in both groups (the placebo group also undergoes the "dose escalation" procedure, but with placebo). |
| Blinding of outcome assessment (detection bias) | Low risk | For the core outcome measures (resolution of steatohepatitis and improvement in liver fibrosis), the trial adopted "centralized read results," and the slides were independently reviewed by "three expert pairs of pathologists" following a "blinded sequence"—pathologists were unaware of the patients' group assignments and baseline biopsy results. |
| Incomplete outcome data (attrition bias) | Low risk | The trial reported complete attrition data: among the 800 enrolled patients, 65 patients (12.2%) in the semaglutide group and 42 patients (15.8%) in the placebo group had missing core pathological outcome data. The attrition rate was low (<20%) in both groups, and the difference between the groups was small (3.6 percentage points), with no evidence of selective attrition (e.g., a significantly higher attrition rate in the experimental group due to adverse reactions).For missing data, the trial used "reference-based multiple imputation informed by observed placebo data," a scientific method for handling missing data that can reduce bias caused by incomplete data. The clear reporting of the imputation method eliminates bias resulting from improper handling of missing data. |
| Selective reporting (reporting bias) | Low risk | The trial was pre-registered on ClinicalTrials.gov (Identifier: NCT04822181). The methods section clearly lists all prespecified outcome measures (2 primary outcomes, 3 secondary outcomes, and safety outcomes), and the results section provides a complete report of all prespecified outcomes. There is no evidence of "unreported prespecified outcomes" or "additional reporting of non-prespecified outcomes." |
| Other bias | NA | NA |
| Overall Assessment | Low risk | Low risk of bias in all domains |

**Supplemental Materials 4. Excluded Studies from databases (n=31)**

| **Author(s)** | **Year** | **Title** | **Reason(s) for Exclusion** |
| --- | --- | --- | --- |
| Naim Alkhouri et al. | 2020 | Safety and efficacy of combination therapy with semaglutide, cilofexor and firsocostat in patients with non-alcoholic steatohepatitis: A randomized, open-label phase II trial | Wrong study design |
| Ahmed I Gad et al. | 2024 | Therapeutic Effects of Semaglutide on Nonalcoholic Fatty hepatic Disease with Type 2 Diabetes Mellitus and Obesity: An Open-Label Controlled Trial | Wrong study design |
| Teruki Miyake et al. | 2022 | Additional Effect of Luseogliflozin on Semaglutide in Nonalcoholic Steatohepatitis Complicated by Type 2 Diabetes Mellitus: An Open-Label, Randomized, Parallel-Group Study | Wrong study design |
| Matthew J Armstrong et al. | 2016 | Glucagon-like peptide 1 decreases lipotoxicity in non-alcoholic steatohepatitis | Wrong study design |
| Matthew Armstrong et al. | 2014 | Effect of liraglutide on adipose insulin resistance and hepatic de-novo lipogenesis in non-alcoholic steatohepatitis: substudy of a phase 2, randomized placebo-controlled trial | The test cycle is short |
| Jinhua Yan et al. | 2019 | Liraglutide, Sitagliptin, and Insulin Glargine Added to Metformin: The Effect on Body Weight and Intrahepatic Lipid in Patients with Type 2 Diabetes Mellitus and Nonalcoholic Fatty hepatic Disease | Wrong study design |
| Manal F Abdelmalek et al. | 2023 | A Phase 2, Adaptive Randomized, Double-blind, Placebo-controlled, Multicenter, 52-week Study of HM15211 in Patients with Biopsy-confirmed Non-alcoholic Steatohepatitis – Study Design and Rationale of HM-TRIA-201 Study | Wrong study design |
| Lin Liu et al. | 2020 | Efficacy of Exenatide and Insulin Glargine on Nonalcoholic Fatty hepatic Disease  in Patients with Type 2 Diabetes Mellitus | Wrong study design |
| Wen Guo et al. | 2020 | Liraglutide or insulin glargine treatments improves hepatic fat in obese patients with type 2 diabetes and nonalcoholic fatty hepatic disease in twenty-six weeks: A randomized placebo-controlled trial | Wrong study design |
| Ning Shao et al. | 2014 | Benefits of exenatide on obesity and non-alcoholic fatty hepatic disease with elevated hepatic enzymes in patients with type 2 diabetes | Wrong study design |
| Manuel Romero-Gómez et al. | 2023 | A phase IIa active-comparator-controlled study to evaluate the efficacy and safety of efinopegdutide in patients with non-alcoholic fatty hepatic disease | Wrong study design |
| Stephen A. Harrison et al. | 2024 | Effect of pemvidutide, a GLP-1/glucagon dual receptor agonist, on MASLD: A randomized, double-blind, placebo-controlled study | Wrong study design |
| Armstrong et al. | 2024 | Liraglutide safety and efficacy in patients with nonalcoholic steatohepatitis (LEAN) | Wrong study design |
| Anne Flint et al. | 2021 | Randomized clinical trial: semaglutide versus placebo reduced hepatic steatosis but not hepatic stiffness in subjects with non-alcoholic fatty hepatic disease assessed by magnetic resonance imaging | Wrong study design |
| Jinhua Yan et al. | 2019 | Liraglutide, Sitagliptin, and Insulin Glargine Added to Metformin: The Effect on Body Weight and Intrahepatic Lipid in Patients with Type 2 Diabetes Mellitus and Nonalcoholic Fatty hepatic Disease | Wrong study design |
| Mohammad S. Kuchay et al. | 2020 | Effect of dulaglutide on hepatic fat in patients with type 2 diabetes and NAFLD: randomized controlled trial (D-LIFT trial) | Wrong study design |
| Mark L. Hartman et al. | 2020 | Effects of Novel Dual GIP and GLP-1 Receptor Agonist Tirzepatide on Biomarkers of Nonalcoholic Steatohepatitis in Patients with Type 2 Diabetes | Wrong study design |
| Arun J Sanyal et al. | 2024 | Triple hormone receptor agonist retatrutide for metabolic dysfunction-associated steatotic hepatic disease: a randomized phase 2a trial | Wrong study design |
| Yuichiro Eguchi et al. | 2015 | Pilot study of liraglutide effects in non-alcoholic steatohepatitis and non-alcoholic fatty hepatic disease with glucose intolerance in Japanese patients (LEAN-J) | Wrong study design |
| David H Ipsen et al. | 2018 | Liraglutide Decreases Hepatic Inflammation and Injury in Advanced Lean Non-Alcoholic Steatohepatitis | The test cycle is short |
| Ling-Yun Zhang et al. | 2020 | Effect of liraglutide therapy on serum fetuin A in patients with type 2 diabetes and non-alcoholic fatty hepatic disease | Wrong study design |
| Wen Guo et al. | 2020 | Liraglutide or insulin glargine treatments improves hepatic fat in obese patients with type 2 diabetes and nonalcoholic fatty hepatic disease in twenty-six weeks: A randomized placebo-controlled trial | Wrong study design |
| Patrick R Kenny et al. | 2010 | Exenatide in the treatment of diabetic patients with non-alcoholic steatohepatitis: a case series | Wrong study design |
| Stephen A Harrison et al. | 2025 | Safety and Efficacy of Efruxifermin in Combination With a GLP-1 Receptor Agonist in Patients With NASH/MASH and Type 2 Diabetes in a Randomized Phase 2 Study | The test cycle is short |
| Naim Alkhouri et al. | 2022 | Safety and efficacy of combination therapy with semaglutide, cilofexor and firsocostat in patients with non-alcoholic steatohepatitis: A randomized, open-label phase II trial | Wrong study design |
| Brandon Havranek et al. | 2025 | Glucagon-like peptide-1 receptor agonists improve metabolic dysfunction-associated steatotic hepatic disease outcomes | Wrong study design |
| Savvoula Savvidou et al. | 2016 | Circulating adiponectin levels in type 2 diabetes mellitus patients with or without non-alcoholic fatty hepatic disease: Results of a small, open-label, randomized controlled intervention trial in a subgroup receiving short-term exenatide | Wrong study design |
| Emmanouil Korakas et al. | 2024 | Semaglutide Concurrently Improves Vascular and hepatic Indices in Patients with Type 2 Diabetes and Fatty hepatic Disease | Wrong study design |
| Sudha S. Shankar et al. | 2024 | Safety and Efficacy of Novel Incretin Co-agonist Cotadutide in Biopsy-proven Noncirrhotic MASH With Fibrosis | The test cycle is short |
| Wu JY et al. | 2025 | Comparative effectiveness of tirzepatide versus bariatric metabolic surgery in adults with metabolic-associated steatotic liver disease and obesity: a multi-institutional propensity score-matched study | Wrong study design |
| [Lin Xiang](https://pubmed.ncbi.nlm.nih.gov/?sort=date&size=50&term=Xiang+L&cauthor_id=39709140) et al. | 2025 | Safety and efficacy of GLP-1/FGF21 dual agonist HEC88473 in MASLD and T2DM: A randomized, double-blind, placebo-controlled study | Wrong study design |
